# Reference Genes for Real-Time PCR Quantification of MicroRNAs and Messenger RNAs in Rat Models of Hepatotoxicity

**DOI:** 10.1371/journal.pone.0036323

**Published:** 2012-05-01

**Authors:** María N. Lardizábal, Ana L. Nocito, Stella M. Daniele, Leonardo A. Ornella, Javier F. Palatnik, Luis M. Veggi

**Affiliations:** 1 IFISE, CONICET-UNR, Rosario, Argentina; 2 Facultad de Ciencias Médicas, Universidad Nacional de Rosario (UNR), Rosario, Argentina; 3 Facultad de Ciencias Bioquímicas y Farmacéuticas, UNR, Rosario, Argentina; 4 CIFASIS, CONICET-UNR-UPCAM, Rosario, Argentina; 5 IBR, CONICET-UNR, Rosario, Argentina; The University of Hong Kong, Hong Kong

## Abstract

Hepatotoxicity is associated with major changes in liver gene expression induced by xenobiotic exposure. Understanding the underlying mechanisms is critical for its clinical diagnosis and treatment. MicroRNAs are key regulators of gene expression that control mRNA stability and translation, during normal development and pathology. The canonical technique to measure gene transcript levels is Real-Time qPCR, which has been successfully modified to determine the levels of microRNAs as well. However, in order to obtain accurate data in a multi-step method like RT-qPCR, the normalization with endogenous, stably expressed reference genes is mandatory. Since the expression stability of candidate reference genes varies greatly depending on experimental factors, the aim of our study was to identify a combination of genes for optimal normalization of microRNA and mRNA qPCR expression data in experimental models of acute hepatotoxicity. Rats were treated with four traditional hepatotoxins: acetaminophen, carbon tetrachloride, D-galactosamine and thioacetamide, and the liver expression levels of two groups of candidate reference genes, one for microRNA and the other for mRNA normalization, were determined by RT-qPCR in compliance with the MIQE guidelines. In the present study, we report that traditional reference genes such as U6 spliceosomal RNA, Beta Actin and Glyceraldehyde-3P-dehydrogenase altered their expression in response to classic hepatotoxins and therefore cannot be used as reference genes in hepatotoxicity studies. Stability rankings of candidate reference genes, considering only those that did not alter their expression, were determined using geNorm, NormFinder and BestKeeper software packages. The potential candidates whose measurements were stable were further tested in different combinations to find the optimal set of reference genes that accurately determine mRNA and miRNA levels. Finally, the combination of MicroRNA-16/5S Ribosomal RNA and Beta 2 Microglobulin/*18S* Ribosomal RNA were validated as optimal reference genes for microRNA and mRNA quantification, respectively, in rat models of acute hepatotoxicity.

## Introduction

Xenobiotic-induced hepatotoxicity is an important cause of liver disease whose comprehension depends on the mechanistic studies of experimental models. The use of whole animals in experimental toxicity is essential to demonstrate that an agent has an adverse effect on the liver in a setting of physiological significance [1]. Hepatotoxicity involves an alteration in the expression of thousands of genes in the liver in response to xenobiotic exposure, a process that has not yet been fully characterized and understood [2].

MicroRNAs are small (19–24 nucleotides) RNA molecules that regulate gene expression at the post-transcriptional level. MicroRNAs are known to participate in numerous physiological and pathological processes and they likely regulate 40% of human genes. Therefore, there is strong interest in analyzing the participation of microRNAs in hepatotoxic events [3], [4]. MicroRNAs typically bind to the 3′-UTR of specific ‘target’ mRNAs [5], [6] and interfere with their translation and/or also accelerate the degradation of the mRNA [7]. Since microRNAs seem to regulate gene expression by a ‘fine-tuning’ mechanism, the study of the participation of microRNAs and their targets in specific physiological or pathological experimental situations depends on a reliable and accurate technique for measuring their expression levels.

In the past several years, reverse transcription quantitative real-time polymerase chain reaction (RT-qPCR) has become the classical technique to measure gene expression due to its accuracy, sensitivity, specificity, reproducibility and robustness [8]–[10]. However, RT-qPCR is a multistage process that includes the extraction of RNA, its reverse transcription and qPCR. Therefore, a rigorous normalization strategy is required to account for the resulting technical variability among samples [11]. The use of reference genes (RGs) as internal controls is the most common method for normalizing qPCR gene expression data. By definition, RG expression levels should be stable across different treatments or cell types in an experiment. The selection of RGs is not trivial and previous studies have demonstrated that a single universal RG is unlikely to exist and perform well for all tissue types or for all physiological, pathological and experimental situations [11], [12]. Moreover, it is highly recommended to use more than one RG in order to produce more reliable data and also because, in this way, it is possible to calculate stability parameters to evaluate the measured RGs in an actual quantification experiment [13].

The identification and validation of the optimal RGs is a crucial process because they will ultimately be responsible for the accuracy of the gene expression determinations reported for the gene of interest. Increasing concern regarding the optimization of normalization methods that use RGs has led to the development of several mathematical algorithms, including BestKeeper [14], geNorm [11] and NormFinder [15], that are aimed at determining the stability of RGs. Because these algorithms are based on the assumption that the candidate RGs are not differentially expressed among groups, the hepatotoxic effects on the relative expression of each candidate RG must be tested prior to the evaluation of their stability. The outputs of the different programs can be compared to obtain a definitive ranking of the RGs using the RankAggreg package [16]. Then, different combinations of the more stable RGs are evaluated for the normalization efficiency, using a strategy that was previously reported [17]. This approach consists on simulating the expression of a hypothetical gene of interest in order to asses the accuracy of transcript quantification resulting with each combination under study. Finally, the selection of the optimal number of RGs for normalization is carried out taking into account the trade-off between the improvement in accuracy and the disadvantage of adding a new gene in the measurement process.

In this report, we thoroughly analyze and select RGs to normalize the RT-qPCR expression data of mRNAs and microRNAs in classical rat models of acute hepatotoxicity. The hepatotoxins administered were acetaminophen (AA), carbon tetrachloride (CT), D-galactosamine (GA) and thioacetamide (TA). We found that several genes that are frequently used to normalize RT-qPCR data must not be applied as RGs in these models. The application of several mathematical methods under the experimental conditions revealed that microRNA 16 (*miR-16*) and *5S* Ribosomal RNA (*5S*) can be used as RGs for microRNAs normalization, whereas Beta 2 Microglobulin (*B2M*) and 18S Ribosomal RNA (*18S*) can be used as RGs for the normalization of mRNA expression data. Our real time qPCR results were obtained in compliance with the global standardization accords reflected in the MIQE guidelines and the RMDL language [18], [19].

## Results

### Assessment of liver injury

**Figure 1:**
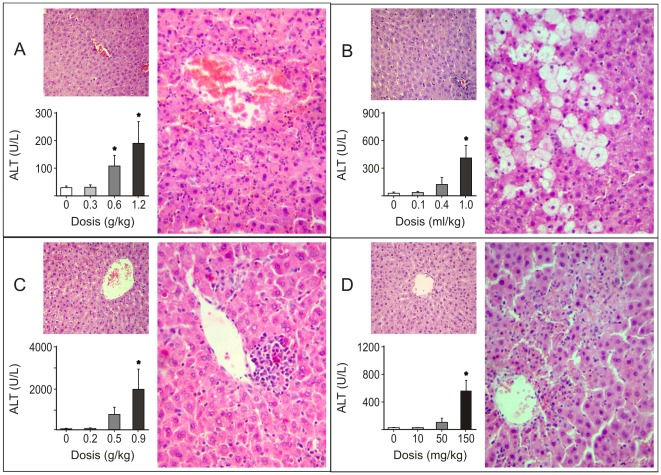
Liver damage assessment in rat models of acute hepatotoxicity. Plasma alanine aminotransferase (ALT) levels and histological examination of the livers from rats after 24 h of intraperitoneal administration of acetaminophen (panel A), carbon tetrachloride (panel B), D-galactosamine (panel C) and thioacetamide (panel D) are shown. The change in the plasma ALT levels in response to increasing doses of each hepatotoxin was tested by a one-way ANOVA followed by the Newman–Keuls test for multiple comparisons. An asterisk (*) indicates a significant difference (p<0.05). Representative histological microphotographs of hematoxylin and eosin stained-liver sections after the administration of highest doses of the hepatotoxins and their respective vehicles (big and small image of each panel, respectively) are shown. The rats were intraperitoneally administered with: acetaminophen (1.2 g/kg body weight) or its vehicle (1% carboxymethyl cellulose, 10 ml/kg body weight); carbon tetrachloride (1 ml/kg body weight) or its vehicle (corn oil, 4.4 ml/kg body weight), D-galactosamine (0.9 g/kg body weight) or its vehicle (saline solution, 6 ml/kg body weight); thioacetamide (150 mg/kg body weight) or its vehicle (saline solution, 6 ml/kg body weight).

The rat models of acute hepatotoxicity for the four hepatotoxins administered in the dose-response protocol were characterized evaluating plasma biochemical markers of liver injury and a histological examination at 24 h post treatment. The alanine aminotransferase activities of the groups treated with AA, CT, GA and TA were significantly increased at the highest doses administered (Figure 1). The changes in the aspartate aminotransferase activities showed almost the same pattern as the alanine aminotransferase activities for all groups (data not shown). A microscopic examination of the livers allowed for the verification of the injury produced with each hepatotoxin and the description of their typical histopathological features. Representative sections of the livers from the control and the chemically treated rats 24 h after the highest dosing are shown in Figure 1. The administration of AA (1.2 g/kg body weight) induced focal necrosis in the centrilobular region with infiltration of neutrophils and lymphocytes. The treatment with CT (1 ml/kg body weight) resulted in moderate to intense hepatocyte necrosis and cytoplasmic vacuolization. The animals treated with GA (0.9 g/kg body weight) presented a mild infiltrate of lymphocytes, macrophages and neutrophils in the centrilobular region, necrosis and apoptotic bodies. Lastly, the TA-treated rats (150 mg/kg body weight) showed a severe infiltration of neutrophils, hepatocyte necrosis and apoptosis. The studies of RG selection were performed on the livers of rats treated with the highest doses of each hepatotoxin comparing to the livers of rats treated with their corresponding vehicle.

### RT-qPCR expression studies of candidate reference genes

The selection of the candidate RGs for microRNAs and for mRNA was performed using two different set of genes (Table 1). In the case of microRNA, the evaluated candidate RGs included *5S*, *miR-16*, MicroRNA 103 (*miR-103*), MicroRNA 191 (*miR-191*), MicroRNA Let7a (*miR-Let7a*), U6 Spliceosomal RNA (*U6*) and Small Nucleolar RNA 48 (*RNU48*). These candidate genes were selected because they are commonly used as endogenous controls and/or because of their relative quantities in liver [20]–[24]. In the case of the RGs for mRNA normalization, the evaluated genes were *18S*, Beta Actin (*ACTB*), Albumin (*ALB*), *B2M*, Cyclophilin A (*CYCA*), Glyceraldehyde-3P Dehidrogenase (*GAPDH*), Hypoxanthine-guanine P-ribosyl Transferase 1 (*HPRT1*) and Succinate Dehydrogenase (*SDHA*), which were selected from the literature and represent those commonly used as normalizers in liver gene expression studies [25]–[29].

**Table 1:** Descriptions of selected candidate reference genes and genes of interest.

Gen Symbol	Gene Name	GenBank or miRbase Acc. N°	Gene Function
**Candidate reference genes for microRNA normalization**			
*5S*	5S Ribosomal RNA	K01594.1	Protein synthesis
*miR-16*	MicroRNA 16	MI0000844	Regulation of apoptosis
*miR-103*	MicroRNA 103	MI0000888	Regulation of neuroblastoma cell migration
*miR-191*	MicroRNA 191	MI0000934	No functionally verified targets
*miR-Let7a*	MicroRNA Let7a	MI0000827	Regulation of cell proliferation
*RNU48*	Small Nucleolar RNA 48	X96648.1	Modification of small nuclear RNAs
*U6*	U6 Spliceosomal RNA	K00784.1	RNA Splicing
**Candidate reference genes for mRNA normalization**			
*18S*	18S Ribosomal RNA	V01270	Protein Synthesis
*ACTB*	Beta Actin	NM_031144.2	Cytoskeletal structural protein
*ALB*	Albumin	NM_134326.2	Major plasma protein
*B2M*	Beta 2 Microglobulin	NM_012512.1	Beta-chain of major histocompatibility complex
*CYCA*	Cyclophilin A	M19533.1	Serine-threonine phosphatase inhibitor
*GAPDH*	Glyceraldehyde-3P-dehidrogenase	NM_017008.3	Glycolysis pathway enzyme
*HPRT1*	Hypoxanthine-guanine P-ribosyl transferase 1	NM_012583.2	Metabolic salvage of purines
*SDHA*	Succinate Dehydrogenase	NM_130428.1	TCA pathway enzyme
**Genes of interest**			
*miR-122*	MIcroRNA 122	MI000891	Regulation of lipid metabolism
*CCNG1*	Cyclin G1	NM_012923.2	Cellular growth

The compliance of the RT-qPCR experiments with the MIQE (Minimum Information for Publication of Quantitative Real-Time PCR Experiments, [http://www.rdml.org/miqe]) guidelines [18] is shown in the MIQE checklist (Table S1). All qPCR results included in this report are available in RDML data format (Real-time PCR Data Markup Language (RDML) [http://www.rdml.org]) [19], including the raw microRNA and mRNA expression data and the experimental and sample annotation files (Data S1). In the following paragraphs we describe relevant information related to the RT-qPCR assays that were carried out. 5S 20 [30]miR-16 [31] 21miR-103 [31] 21 miR-191 [31] 21 miR-Let7a [31] 21 RNU48 [31] 21 U6 22 [32] 18S ACTB 23 [33] ALB 24 [34] B2M 25 [35] CYCA 26 [36] GAPDH HPRT1 27 [26] SDHA 27 [26]

RNA was purified from liver samples of control rats and rats treated with the different hepatotoxins. Only high-quality RNA samples were included in this study, according to the quality specifications established in the item “RNA purification" of the Materials and Methods section. The amplification efficiencies for all evaluated candidate RGs and the genes of interest ranged from 91 to 104% (Table 2), with correlation coefficients of standard curves ranging from 0.983 to 0.999. Gene-specific amplification was confirmed by a single peak in the melting-curve analysis and a single band of the expected size on a 2% agarose gel stained with ethidium bromide (for a representative trace, see Figure S1).

**Table 2:** Descriptions of gene-specific real-time PCR assays.

Gen Symbol	Primer Sequences (5′-3′)	Am (bp)	Tm (°C)	E (%)	Ref
**Candidate reference genes for microRNA normalization**					
*5S*	RT: AGCCTACAGCACCCGGTATT	40	81.6	94	[30]
	F: GCCCGATCTTGTCTGATCTC				
	R: CCTGACCCTGCTTAGCTTCC				
*miR-16*	RT: GTCTCCTCTGGTGCAGGGTCCGAGGTATTCGCACCAGAGGAGACCGCCAA	50	83.5	91	[31]
	F: CAGCCTAGCAGCACGTAAAT				
	R: GAGGTATTCGCACCAGAGGA				
*miR-103*	RT: GTCTCCTCTGGTGCAGGGTCCGAGGTATTCGCACCAGAGGAGACTCATAG	51	79.8	104	[31]
	F: TACGCAGCAGCATTGTACAG				
	R: GAGGTATTCGCACCAGAGGA				
*miR-191*	RT: GTCTCCTCTGGTGCAGGGTCCGAGGTATTCGCACCAGAGGAGACCAGCTG	49	80.0	104	[31]
	F: CACCAACGGAATCCCAAA				
	R: GAGGTATTCGCACCAGAGGA				
*miR-Let7a*	RT: GTCTCCTCTGGTGCAGGGTCCGAGGTATTCGCACCAGAGGAGACAACTAT	50	81.5	96	[31]
	F: CGCGCTGAGGTAGTAGGTTG				
	R: GAGGTATTCGCACCAGAGGA				
*RNU48*	RT: GTCTCCTCTGGTGCAGGGTCCGAGGTATTCGCACCAGAGGAGACGGTCAG	65	88.5	93	[31]
	F: TCTGAGTGTCTTCGCTGACG				
	R: GAGGTATTCGCACCAGAGGA				
*U6*	RT: AACGCTTCACGAATTTGCGT	93	85.7	94	[32]
	F: CTCGCTTCGGCAGCACA				
	R: AACGCTTCACGAATTTGCGT				
**Candidate reference genes for mRNA normalization**					
*18S*	RT: GAGCTGGAATTACCGCGGCT	159	87.8	97	a
	F: AAACGGCTACCACATCCAAG				
	R: TTGCCCTCCAATGGATCCT				
*ACTB*	F: ATTGCTGACAGGATGCAGAA	109	86.7	104	[33]
	R: TAGAGCCACCAATCCACACAG				
*ALB*	F: GATGCCGTGAAAGAGAAAGC	196	89.2	93	[34]
	R: CGTGACAGCACTCCTTGTTG				
*B2M*	F: ACATCCTGGCTCACACTGAA	109	87.1	94	[35]
	R: ATGTCTCGGTCCCAGGTG				
*CYCA*	F: AGCACTGGGGAGAAAGGATT	248	87.8	97	[36]
	R: AGCCACTCAGTCTTGGCAGT				
*GAPDH*	F: GTATCGGACGCCTGGTTAC	128	87.1	93	a
	R: CTTGCCGTGGGTAGAGTCAT				
*HPRT1*	F: GCTGAAGATTTGGAAAAGGTG	157	87.7	92	[26]
	R: AATCCAGCAGGTCAGCAAAG				
*SDHA*	F: AGACGTTTGACAGGGGAATG	160	89.8	97	[26]
	R: TCATCAATCCGCACCTTGTA				
**Genes of interest**					
*miR-122*	RT: GTCTCCTCTGGTGCAGGGTCCGAGGTATTCGCACCAGAGGAGACCAAACA	50	83.4	97	[31]
	F: GGCTGTGGAGTGTGACAATG				
	R: GAGGTATTCGCACCAGAGGA				
*CCNG1*	F: AGTCTTAAGGGACGTCAGGAG	119	86.1	92	a
	R: GCTGAGGAGCTACCCACATT				

Am: amplicon size. bp: numer of base pairs. E: Assay efficiency. Tm: melting temperature. Ref: references. RT: retro-transcription primer. R: Reverse primer. F: Forward primer. a: Primers were designed using Primer3 software.

**Figure 2:**
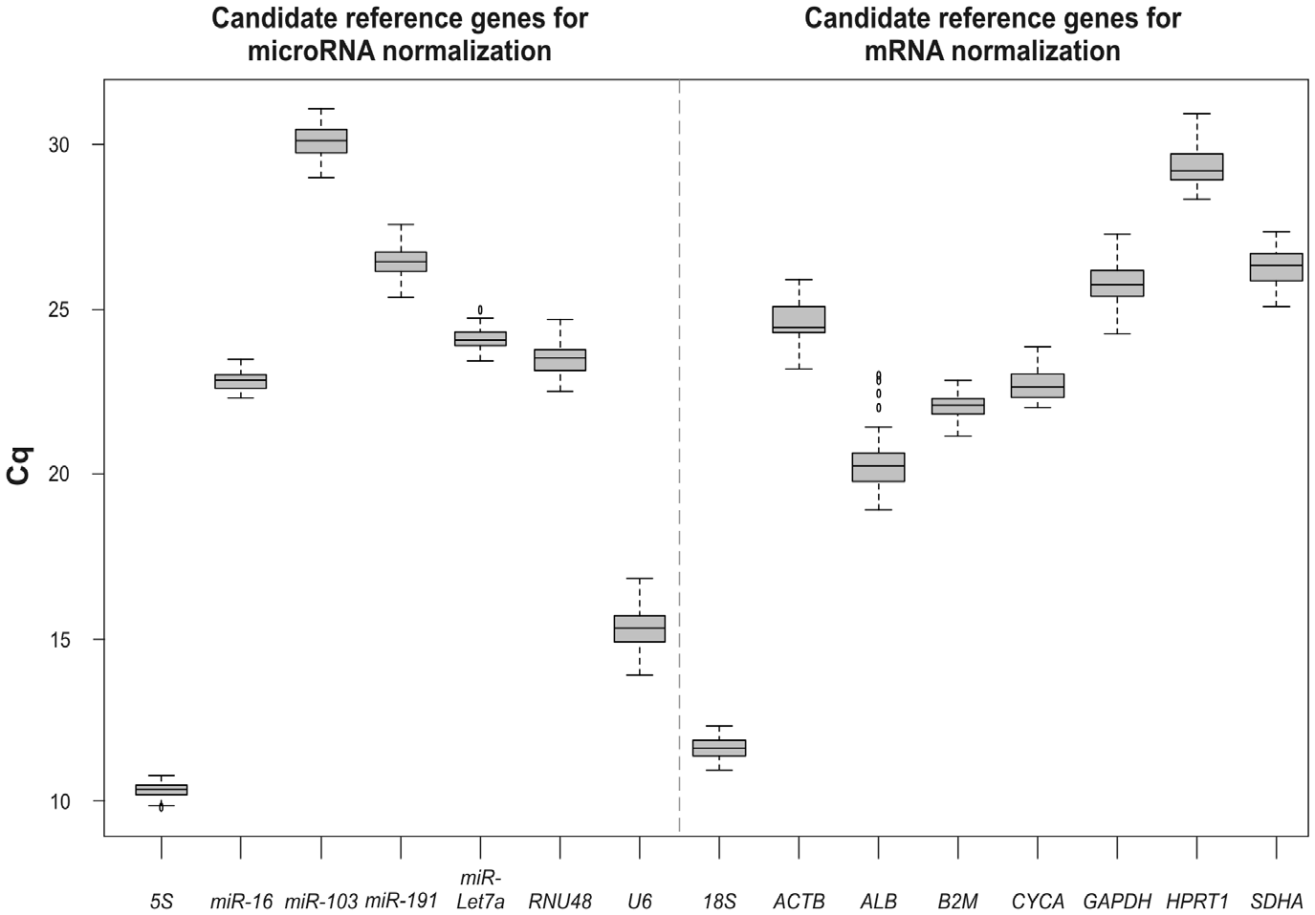
Boxplot of RT-qPCR quantification cycles values of candidate reference genes. Boxplot of quantification cycles (Cq) values for each reference gene for microRNA and mRNA normalization in all liver samples assessed (n = 35) which belong to rats treated with each one of the four hepatotoxins studied and their respective control animals administered with vehicle. A line across the box depicts the median. The box indicates the 25% and 75% percentiles. Whiskers represent the maximum and minimum values, circles represent outliers. The livers were evaluated 24 h after rats were intraperitoneally administered with: acetaminophen (1.2 g/kg body weight) or its vehicle (1% carboxymethyl cellulose, 10 ml/kg body weight); carbon tetrachloride (1 ml/kg body weight) or its vehicle (corn oil, 4.4 ml/kg body weight), D-galactosamine (0.9 g/kg body weight) or its vehicle (saline solution, 6 ml/kg body weight); thioacetamide (150 mg/kg body weight) or its vehicle (saline solution, 6 ml/kg body weight).

RT-qPCR was performed to evaluate the expression patterns of the candidate RGs in the liver of the control rats and the rats treated with different hepatotoxins (Figure 2). The average standard deviation within duplicates of all samples studied was 0.10 cycles. The expression levels displayed a wide range of quantification cycle (Cq) values ranging from 9.82 to 31.06 for the microRNA RGs and from 11.09 to 30.93 for the mRNA RGs. *MiR*-*103* and *HPRT1* showed relatively low expression (Cq>28.37), whereas *5S* and *18S* showed relatively high expression (Cq<12.45) in both groups of RGs. Comparing the Cq of the RGs in each group, *5S* and *18S* showed the least variability, and *U6* and *ALB* displayed the most variability. As the 5S and 18S transcripts accumulated at high levels, we confirmed that their expression data were included in the quantitative dynamic range of our RT-qPCR assays (Figure S2).

### Testing for expression differences in reference genes associated with the exposure to hepatotoxins

**Figure 3:**
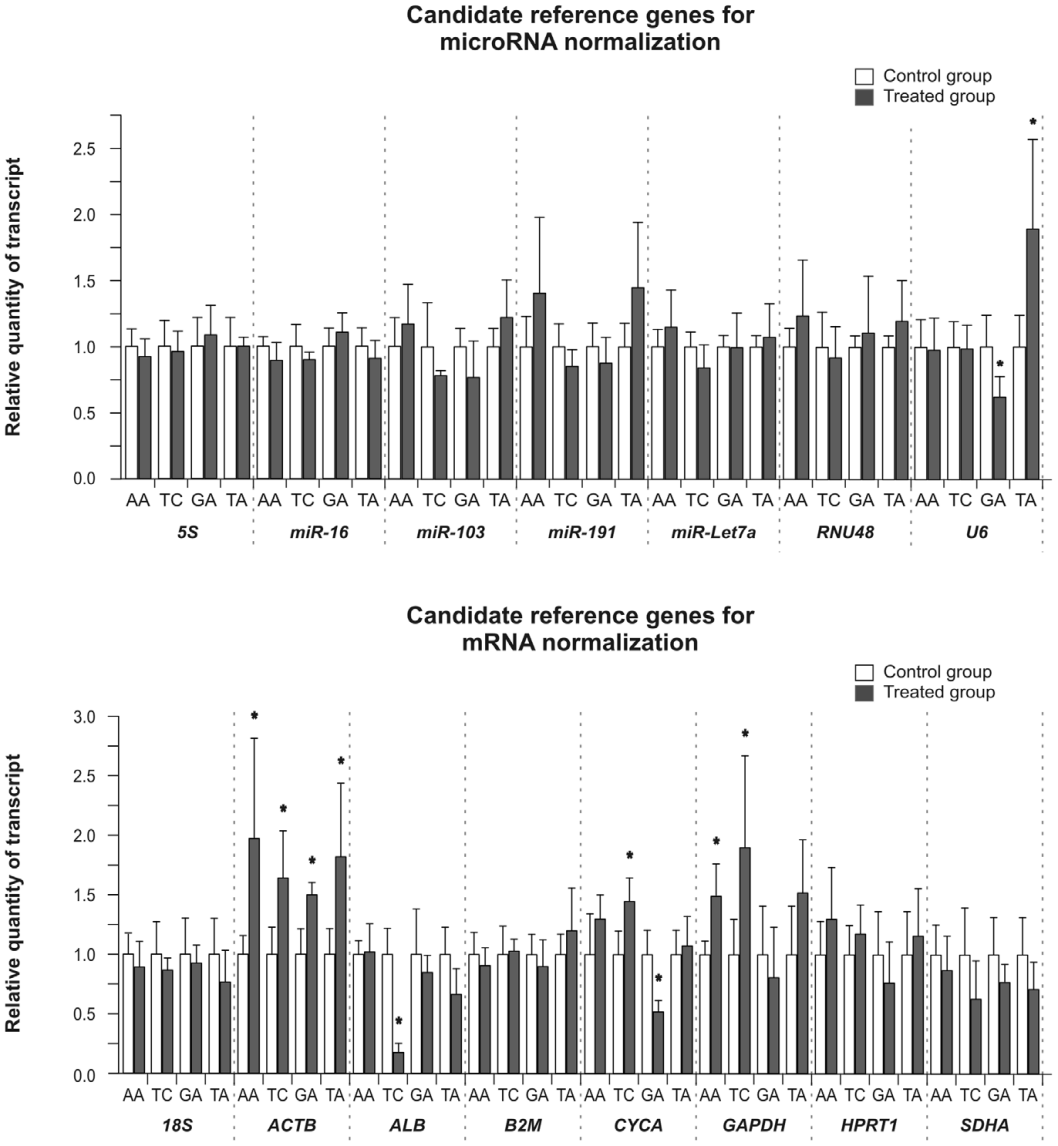
Effect of rat exposure to hepatotoxins on liver expressions of candidate reference genes. The expression of candidate reference genes for microRNA and mRNA normalization in the liver of rats treated with the hepatotoxins and their respective vehicles are shown. The livers were evaluated 24 h after rats were intraperitoneally administered with: acetaminophen (AA, 1.2 g/kg body weight) or its vehicle (1% carboxymethyl cellulose, 10 ml/kg body weight); carbon tetrachloride (CT, 1 ml/kg body weight) or its vehicle (corn oil, 4.4 ml/kg body weight), D-galactosamine (GA, 0.9 g/kg body weight) or its vehicle (saline solution, 6 ml/kg body weight); thioacetamide (TA, 150 mg/kg body weight) or its vehicle (saline solution, 6 ml/kg body weight). The bars represent the means of the relative quantity of transcript ± SD of 5 animals treated with hepatotoxins or their respective vehicle. An asterisk (*) indicates a significant expression difference with p<0.05 using Student's t-test of the log-transformed data between the control and treated groups.

The basic requirement of a candidate gene to be used for normalization purposes is unvarying expression in the respective study groups. Thus, specific validation is necessary for each candidate RG prior to its expression stability study. The Student's t-test (t-test) was used to compare the log transformation of the relative quantity of transcript (RQ) of the candidate RG between the samples from the control and the treated groups for each hepatotoxin (Figure 3). When the obtained p values were between 0.1 and 0, the experiments were repeated with a new set of animals to confirm the results (data not shown). Significant differences in the RQ (p<0.05) with respect to the control groups were observed for *ACTB* and *GAPDH* in the AA-treated livers, for *ACTB*, *ALB*, *CYCA* and *GAPDH* in the livers from the TC-treated rats, for *U6*, *ACTB* and *CYCA* in the livers of the GA group and for *U6* and *ACTB* in the livers from the TA-treated rats. These altered expression observed demonstrate that these genes are unsuitable for the normalization of RT-qPCR data. Therefore, these genes were excluded from subsequent calculations.

### Rank of reference genes according to their expression stability

From a theoretical perspective, the group of genes, *5S*, *miR-16*, *miR-103*, *miR-191*, *miR-Let7a* and *RNU48*, and the group of genes, *18S*, *B2M*, *HPRT1* and *SDHA*, can be used for microRNA and for mRNA expression data normalization in our models of acute hepatotoxicity, respectively, because they showed no expression differences. However, to find the best RGs for an accurate normalization strategy, it is necessary to determine the most stably expressed gene, i.e., the RGs with minimal biological variance. Because no unique criterion exists to study this issue, we decided to apply the three most frequently used programs: BestKeeper, geNorm and NormFinder. The RankAggreg package was subsequently used to obtain a definitive stability ranking by compiling the output of each program.

The results are summarized in Table 3. With BestKeeper, the stability (SD, standard deviation) and the relationship to the BestKeeper index (Pearson correlation coefficient r and p values) are the two most important criteria for evaluating the stability of RGs. This program uses a pairwise correlation analysis for all pairs of candidate genes based on the raw Cq values and calculates the geometric mean of the best suited candidates to establish the BestKeeper index. Based on the inspection of the SD, Best-Keeper revealed an overall stability in gene expression (SD<1) for all candidate genes (Data S2). All RGs, except for *RNU48*, were significantly correlated to the BestKeeper index (p<0.05). The Pearson correlation coefficient of each gene gave the highest stability expression to *miR-16* and *miR-191* for microRNA studies and *SDHA* and *18S* for mRNA studies (Data S2).

**Table 3:** Ranking of candidate reference genes according to their stability value.

Stability order	Bestkeeper	geNorm	NormFinder	Consensus
**Candidate reference genes for microRNA normalization**				
1	*miR-16*	*miR-16/5S*	*miR-16*	*miR-16*
2	*miR-191*		*5S*	*5S*
3	*5S*	*miR-Let7a*	*miR-Let7a*	*miR-Let7a*
4	*miR-103*	*miR-191*	*miR-191*	*miR-191*
5	*miR-Let7a*	*miR-103*	*miR-103*	*miR-103*
6	*RNU48*	*RNU48*	*RNU48*	*RNU48*
**Candidate reference genes for mRNA normalization**				
1	*SDHA*	*B2M/18S*	*B2M*	*B2M*
2	*18S*		*18S*	*18S*
3	*B2M*	*SDHA*	*SDHA*	*SDHA*
4	*HPRT1*	*HPRT1*	*HPRT1*	*HPRT1*

Each column refers to a gene stability ranking computed by one statistical tool, using all gene expression values measured for each candidate reference gene. The stability measurements produced by geNorm, NormFinder and BestKeeper were combined to establish a consensus rank of the genes applying the RankAggreg package.

The geNorm program calculates the M stability value of a gene based on the average pairwise variation between all studied genes. A high gene expression variability results in high M values and indicates low expression stability. The M values of the evaluated genes were all under 1.5, which indicates that the expressions of the different candidate genes are relatively stable. The highest-ranked genes were *miR-16* and *5S* for microRNA and *B2M* and *18S* for mRNA expression studies (Data S2).

NormFinder employs a model-based approach that, in addition to the overall expression level variation, also takes into account the intra- and intergroup variation of the candidate normalization genes to evaluate the expression stability. Using NormFinder, the top-ranked RGs were *miR-16* and *5S* in the group of candidate RGs for microRNA and *B2M* and *18S* in the group of candidate RGs for mRNA (Data S2).

A comparison of the rankings produced by the three approaches revealed differences as a consequence of the different algorithms used by each program (Table 3). The stability measurements produced by geNorm, NormFinder and BestKeeper were combined to establish a consensus rank of the genes applying the RankAggreg package. Specifically, the BruteAggreg function of this package performs an aggregation of ordered lists based on the ranks using a brute-force algorithm, i.e. generating all possible ordered lists and finding the list with the minimum value of the objective function. To generate all possible ordered lists, the permutation function from the tool package was used, and an unweighted rank aggregation was performed. We were able to use this function because we had a number of candidate RGs less than 10 in both cases. The input for this statistical package was a matrix of rank-ordered genes according to the different stability measurements previously computed. Because geNorm gives the same M stability value for the two most stable genes, two consensus lists of RGs were constructed, altering the position of the two most stable genes in the geNorm list. The Spearman footrule function was applied to calculate the “distance" among ordered lists. If the two possible analyses (with each one of the geNorm possibility) results in different rankings, the consensus ranking with the lowest score was chosen. Subsequently, using the BruteAggreg function, the top-ranked genes were *miR-16*, *5S* and *miR-Let7a* in the group of candidate RGs for microRNA and *B2M*, *18S* and *SDHA* in the group of candidate RGs for mRNA (Data S2).

### Determination of the optimal number of reference genes

**Figure 4:**
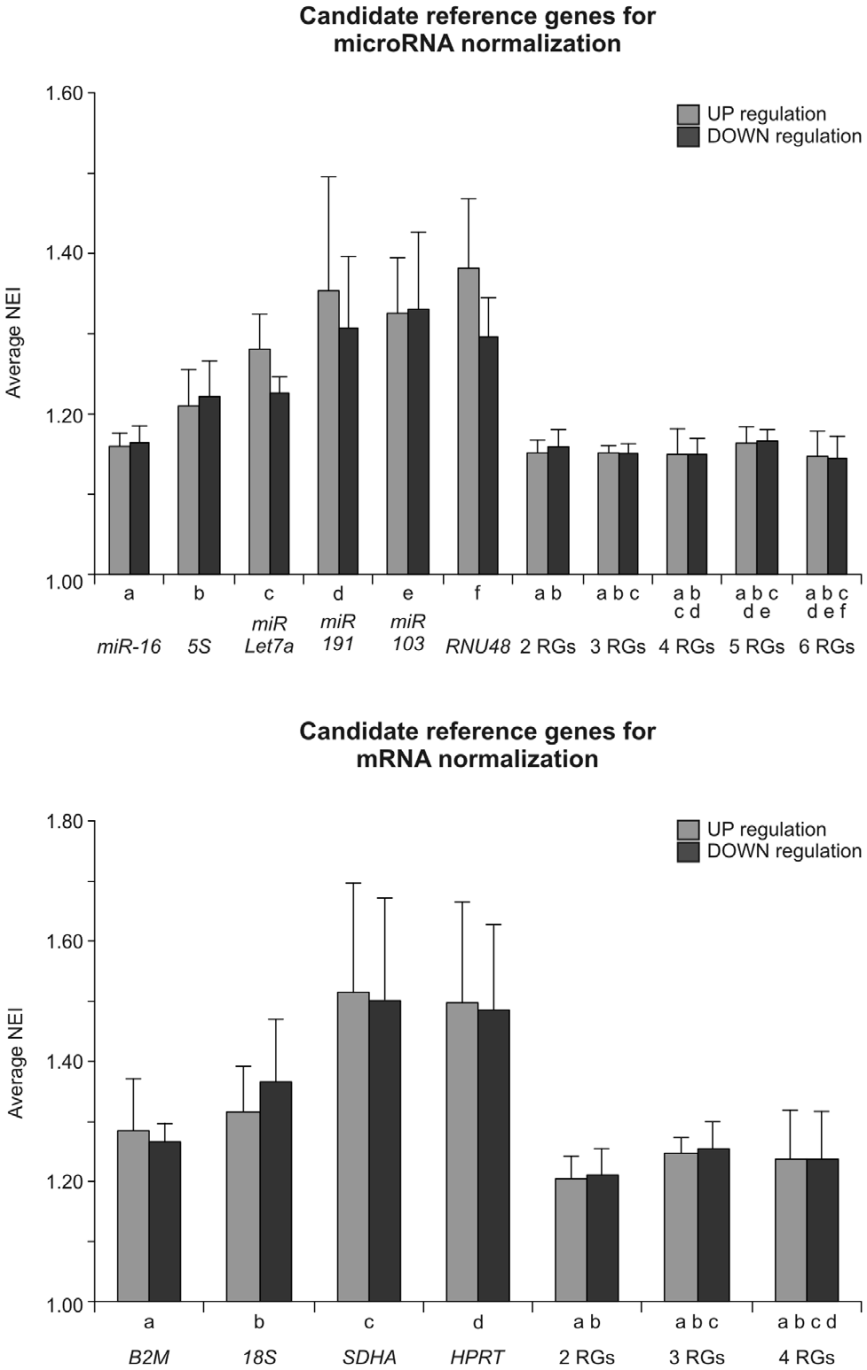
Study of the normalization efficiency of candidate reference genes. The average normalization efficiency index (NEI) of the four treatments for each microRNA or mRNA normalization method and their corresponding standard deviation are shown in up- and down-regulation situations. For each treatment with a specific hepatotoxin, we defined the NEI value as the minimum fold up- or down-regulation of the simulated expression of a hypothetical gene needed to observe a significant difference (t test, p = 0.05, n = 5) using different methods of normalization. The liver expression value of one or combination of reference genes (RGs) was used to normalize the expression of the hypothetical gene. The liver expression of RGs were assessed 24 h after rats were intraperitoneally administered with: acetaminophen (AA, 1.2 g/kg body weight) or its vehicle (1% carboxymethyl cellulose, 10 ml/kg body weight); carbon tetrachloride (CT, 1 ml/kg body weight) or its vehicle (corn oil, 4.4 ml/kg body weight), D-galactosamine (GA, 0.9 g/kg body weight) or its vehicle (saline solution, 6 ml/kg body weight); thioacetamide (TA, 150 mg/kg body weight) or its vehicle (saline solution, 6 ml/kg body weight).

The optimal number of RGs was determined evaluating the normalization efficiency resulting of adding RGs stepwise according to the stability ranking. The evaluation of the normalization efficiency involves the estimation of the accuracy in the quantification of a hypothetical gene of interest through the determination of the minimum expression difference detectable between experimental groups for each combination of RGs. The expression of the hypothetical gene is simulated in the control and the treated groups so that, after the arbitrary expression value is normalized with a specific combination of RGs, the minimal statistically significant difference that could be observed between them can be determined. Although our simulation of expression levels for the hypothetical gene of interest did not include any variability among biological samples belonging to the same experimental group, this strategy results very useful to compare the different normalizers. In order to simplify the application and interpretation of the method to determine the number of RGs described in this paragraph, we defined a new parameter named the normalization efficiency index (NEI). This value is the minimum fold change (for up- or down-regulation) in the normalized relative quantity of transcript (NRQ) of a hypothetical gene of interest that gives statistically significant differences with the t-test (p<0.05), using the candidate RGs under study. The lower the NEI value is, the more efficient is the normalization method evaluated, considering the up- or down-regulation in one toxin-treated group. In our case, we calculated the average NEI value (including the NEI values for AA, CT, GA and TA) for situations of up- and down-regulation and used it to evaluate the different normalization methods and to establish the optimal number of RGs to be used. Figure 4 shows that, when considering the normalization method with only one RG, *miR-16* and *B2M* are the most efficient normalizers for their respective microRNA and mRNA RG groups. This similar tendency is observed when the NEI value is analyzed for each individual hepatotoxin (Figure S3).

The use of two or more RGs as normalizers has been highly recommended because the use of multiple RGs allows for the evaluation of the stability of these RGs in each experiment [13]. The definition of the number of genes to be used for normalization (beginning from two) must involve the valuation of the trade-off between the benefits in accuracy when introducing a new RG and the increased labor and costs of the experiment. From the data displayed in Figure 4 it can be seen that the use of three or more RGs does not introduce any significant advantage in the normalization efficiency, as evaluated by the NEI values in both groups of RGs, considering both senses of modification. An evaluation of the NEI values for the individual treatments demonstrates that the inclusion of a third RG does not lead to a normalization improvement in any of the four studied hepatotoxins, considering both groups of RGs (Figure S3). Therefore, we recommend the use of *miR-16*/*5S* and *B2M*/*18S* for microRNA and mRNA expression normalization data, respectively.

Vandesompele et al. [11] defined a variable “V" that is used to evaluate the improvement of the normalization method that results from the sequential addition of RGs. “V_n/n+1_" represents the pairwise variation in the normalization factor for n RGs and the normalization factor of n+1 RGs. As a consequence, it reflects the effect of the normalization factor stability with the inclusion of an additional gene. According to the original publication of the geNorm developers, the additional RG included is considered not to improve the normalization accuracy significantly if the pairwise variation resulting from the evaluation of n genes and n+1 genes is below a cut-off value of 0.15. If we apply this parameter in our resulting consensus rank stability of candidate RGs for microRNA, the pairwise variation calculations are V_2/3_ = 0.114, V_3/4_ = 0.122, V_4/5_ = 0.085 and V_5/6_ = 0.080. We found a complete coincidence between the two criteria, indicating that two RGs, *miR-16* and *5S*, are the best normalizers for microRNA expression studies. In the case of the candidate RGs for mRNA, we observed a discrepancy in the number of recommended genes. Considering the V value criterion, the use of three RGs is recommended (V_2/3_ = 0.174 and V_3/4_ = 0.140). However, taking into account that others have reported that there was no significant improvement with the addition of a new RG with “V" values even more than 0.20 [37]–[40] and also considering the technical and economic advantages of using one less RG, we confirm the recommendation of using *B2M* and *18S* as the selected mRNA normalization method.

#### Confirmation of the altered expression of candidate reference genes

To confirm the alteration of the candidate RGs in the treated rat livers reported above, we normalized their RQ levels using the method validated in this report. The normalization factor was calculated as the geometric mean of the combination of two genes (*miR-16*/*5S* for microRNAs and *B2M*/*18S* for mRNAs). The statistical significance of the differential log-transformed expression levels between the groups was assessed by the t-test with a p<0.05. The NRQ value of each gene is presented as mean ± SD (n = 5). The NRQ of *U6* diminished in the livers of the rats treated with GA with respect to the vehicle-treated rats (C 1.00±0.16 vs. GA 0.42±0.04, p<0.001) and increased in the livers of the rats treated with TA (C 1.00±0.05 vs. TA 1.45±0.21, p<0.001). With respect to the candidate RGs for mRNA studies, we observed an increased NRQ of *ACTB* in the liver of AA-treated rats (C 1.00±0.17 vs. AA 1.84±0.78, p<0.05), in the liver of CT-treated rats (C 1.00±0.10 vs. CT 1.74±0.45, p<0.001), in the liver of GA-treated rats (C 1.00±0.17 vs. GA 1.86±0.31, p<0.001), and in the liver of TA-treated rats (C 1.00±0.29 vs. TA 2.56±0.43, p<0.001). *ALB* was another candidate RG evaluated that showed a markedly altered NRQ in the livers of the rats treated with CT (C 1.00±0.22 vs. CT 0.35±0.07, p<0.001), whereas such NRQ changes were observed for *CYCA* in the livers of the CT-treated rats (C 1.00±0.14 vs. CT 1.99±0.31, p<0.001) and the GA-treated rats (C 1.00±0.09 vs. GA 0.62±0.21, p<0.05). Lastly, *GAPDH* showed an increased expression level in the livers of the rats treated with AA (C 1.00±0.34 vs. AA 2.55±0.74, p<0.005) and with CT (C 1.00±0.16 vs. CT 1.64±0.36, p<0.005). To test the quality of these expression studies, the values of the parameter M (introduced in the geNorm program description), and the coefficient of variation of the NRQs of the RGs were calculated for all of the experiments presented in this section; the results in all cases were lower than the cut-off values (0.5 and 25%, respectively) established in a previous report [13].

### Evaluation of genes of interest expression during acute hepatotoxicity

Previous microarray analysis have shown that MicroRNA 122 (miR-122) diminishes its expression in AA-treated mouse livers 24 hours after toxic administration [41]. In order to evaluate some of the normalization methods analyzed in this report, we studied the liver expression of miR-122 and its validated mRNA target, Cyclin G1 (CCNG1) [42], in AA treated rats, using the same experimental design used to confirm the altered expression of RGs.

We determined the expression level of miR-122 by RT-qPCR using the two selected RGs (miR-16/5S) in the liver of AA-treated rats and, as expected, we observed a decrease in miR-122 levels (C 1.00±0.18 vs. AA 0.62±0.16, p<0.01). When we normalized the miR-122 expression levels using RNU48, a candidate microRNA RG with a lower expression stability, we still observed a significant change in AA-treated rats (C 1.00±0.24 vs. AA 0.49±0.21, p<0.01). However, there is a loss in accuracy of the normalization, evidenced by the increase in the standard deviation, showing that miR-16/5S performed better than RNU48. These differences in the efficiency of normalization using the miR-16/5S combination or RNU48 are in agreement with the previous analysis using the NEI. Furthermore, considering that the analysis using an hypothetical gene of interest is more specific because it does not include the intra-groups variability intrinsic of a real gene, the NEI value provides an advantage in the evaluation of normalization methods.

When we used the two RGs that we selected, B2M/18S, as normalization method to study the CCNG1 transcript levels in livers of AA-treated rats, we observed a significant increase (C 1.00±0.26 vs. AA 1.95±0.67, p<0.01). This observation is in accordance with the diminished expression of its negative regulator miR-122 in AA-treated livers and is in agreement with previous data showing an inverse correlation between miR-122 and CCNG1 expression [42]. Interestingly, the normalized liver expression levels of CCNG1 would not have changed if the RGs used were ACTB (C 1.00±0.36 vs. AA 0.99±0.56, p<0.386) or GAPDH (C 1.00±0.30 vs. AA 1.29±0.78, p<0.253). These observations emphasize the importance of carrying out pilot experiments to evaluate the possible alteration of candidate RGs expression levels between the groups under study.

## Discussion

The relative quantification of gene expression by Real-Time qPCR has become one of the major methods for studying microRNA and mRNA levels in tissues or cell cultures. However, the performance of this technique is strongly dependent on an adequate normalization strategy through the selection of stably expressed RGs. It has been demonstrated that classical RGs altered their expression under different experimental situations [11], [12]. This is the first report where suitable RGs for expression studies of microRNAs and mRNAs are identified in rat models of acute hepatotoxicity.

AA, CT, GA and TA are standard hepatotoxins used in experimental toxicity studies for the elucidation of the general mechanisms of liver injury and compensatory tissue repair, testing hepatoprotective treatments and identifying potential biomarkers [1], [43]. The validation of the acute hepatotoxicity models through the verification of the damaged induced is in accordance with the classical reports. In our case, we confirmed the liver responses to the exposure of the hepatotoxins (AA, CT, GA and TA) through doses-response studies evaluating the plasma transaminase levels and histopathology examination.

**Figure 5:**
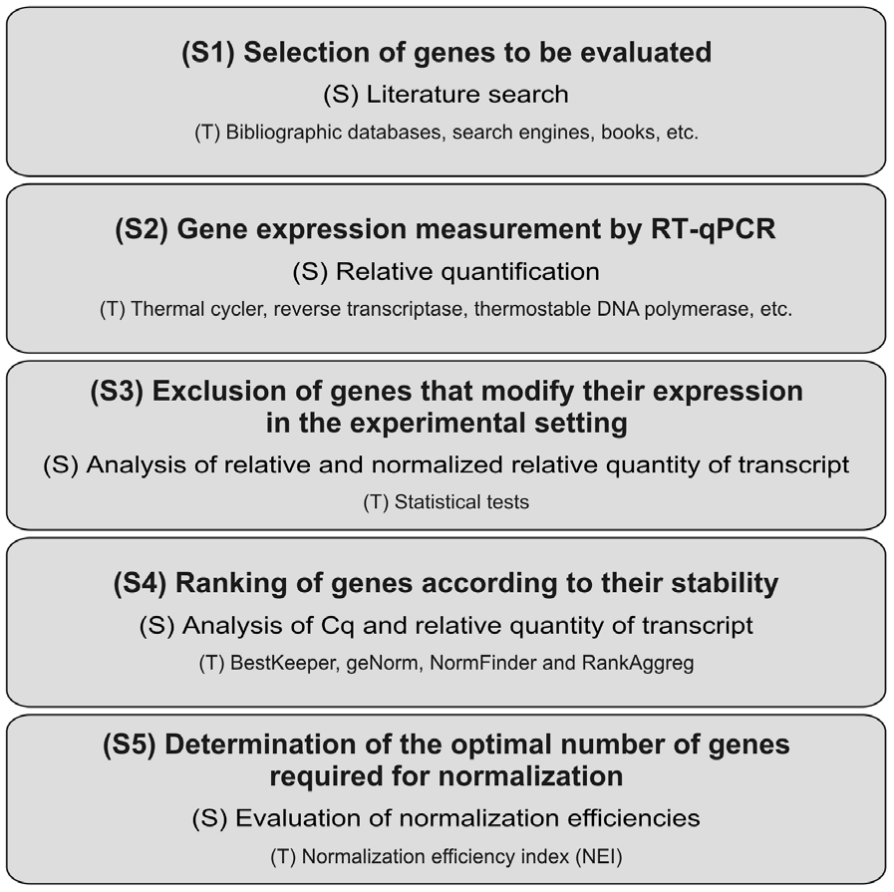
Workflow for reference gene selection. All the steps (S+number) followed for reference gene selection in this report are shown. The strategy (S) applied and the tools (T) used in each step are mentioned in each corresponding panel.

In these rat acute hepatotoxicity models, we selected the optimal RGs according to an analysis of five steps: 1) the selection of the candidate RGs to be evaluated; 2) gene expression measurement by RT-qPCR; 3) the exclusion of the candidate RGs that modify their expression in the experimental setting; 4) the establishment of the expression stability ranking within the remaining group of RGs; and 5) the determination of the number of RGs to be used (Figure 5). Although there are precedents from different reports that use some of these steps, our complete workflow represents an original strategy to select RGs in different experimental designs and models. Some of the statistics tests used in this study (e.g., the t-test and Pearson correlation analyses of BestKeeper) require normally distributed data. Specifically, the qPCR data (RQ and NRQ scale) are nonlinear, and they typically suffer from a heterogeneity of variance across biological replicates, both within treatments and across treatments, which can usually be accounted for applying a log transformation [14], [44]. We confirmed this assertion in our experimental setting verifying the assumption of the normality of the log-transformed dataset of the RQ level of the RGs (for the microRNA and mRNA normalization) that do not modify their expression by the Shapiro-Wilk test (Table S2).

All candidate RGs for microRNA and mRNA normalization were selected based on their known expression in liver tissues, on previous uses as RGs in liver studies and considering that they belong to different functional classes to reduce the chance that the genes might be co-regulated. The demonstration that the candidate RGs do not exhibit modified RQ levels in the livers of the rats treated with hepatotoxins was validated using the t-test. We observed a modification of RQ expression in *ALB*, *CYCA* and *GAPDH* in the CT-treated rats, a change in *GAPDH* RQ expression in the rats treated with AA, an RQ alteration of *U6* and *CYCA* in the GA-group, a change in the RQ of *U6* in the TA-group and, lastly, a modified *ACTB* expression in the livers of all treated groups. All these results were further confirmed testing the statistically significant differences in the NRQ values that were calculated using the optimal RGs proposed in this report as normalizers.

Previous hepatotoxicity studies have described similar alterations of *GAPDH* and *ACTB* associated to AA and CT exposure, respectively. Heinloth et al. [45] found a significant up-regulation of *GAPDH* expression following the 24 and 48 h exposure of rats to a high dose of AA (1500 mg/kg). Additionally, Armendariz-Borunda et al [46] found an approximately two fold increase in the *ACTB* transcript 24 h after CT treatment in rats. These agreements indicate that the liver cells in our experimental setting are responding to the exposure of the hepatotoxins at the level of gene expression. Therefore, this represents another important validation of our models of acute hepatotoxicity, together with the impairment of plasma toxicity markers and the histological damaged observed.

The use of RGs in the normalization procedure that display modifications in their expression between experimental groups could result in serious errors in gene expression studies, which could lead to incorrect conclusions. Despite this, only few previous reports have evaluated this possibility [26], [38], [47]–[49]. In this study, several candidate RGs (among them, the conventional *GAPDH* and *ACTB*) showed altered expression in the acute hepatotoxicity models studied. A similar situation was found in different studies evaluating candidate RGs for mRNA normalization: in a cytotoxicity study using AA [49] and in dioxin-treated rats [26], it was observed that about 40 and 50% of the candidate RGs changed their expressions in response to the hepatotoxin, respectively. These findings highlight the importance of testing the alteration of expression of RGs in toxicity studies.

The overall expression stability is a further major criterion in the selection of the best candidate RG. Different algorithms specifically developed for RGs evaluation and selection were used, e.g., based on repeated RG pairwise correlation and regression analysis [14], ranking and stepwise elimination of the least stable gene [11], or statistical linear mixed-effects modeling [15] of the respective experimental data. Altogether, there were strong similarities among the different programs, but the coincidence in assigning the genes with the highest and lowest score was not absolute. The different rankings generated by the three software packages were compared to obtain a consensus stability order of the RGs using the RankAggreg package of R project [16]. The RankAggreg package has the ability to combine lists obtained from different sources, which may not otherwise be directly comparable; recently, it was introduced to compare the output of RG selection software [50]. In this study, we were able to produce the consensus list provided in Table 3.

An efficient normalization methodology enables the gathering of reproducible and biologically relevant RT-qPCR data correcting non-biological sample-to-sample variations that could be introduced by protocol-dependent inconsistencies. Ideally, the main source of variability in gene expression that is observed is a consequence of the treatment applied to the samples, resulting in the possibility of detecting smaller biologically induced differences through statistical analyses. We defined the NEI value as the minimum fold up- or down-regulation of the NRQ needed to observe a significant difference (t-test, p = 0.05) using a particular normalization method (a specific combination of RGs in both number and identity) in a specific hepatotoxin treatment (AA or CT or GA or TA). Strategies using the normalization of a simulated hypothetical gene expression dataset to evaluate normalization methods were used in previous reports [17]. When we analyzed the ranking of RGs according to both their stability (Table 3) and their average NEI value for up- and down-regulation when the normalization is to a single RG (Figure 4), we observed the lowest NEI values for the best ranked RGs on the expression-stability scale and the highest NEI values for the least stable RGs. This observation validates the procedure proposed in this work to evaluate the stability of the putative RGs.

The minimum number of RGs recommended to use in a normalization method is two, as it is possible to obtain more accurate data and to test the stability of the chosen RGs in an actual quantification experiment [13]. When we analyze the efficiency of normalization with RGs added stepwise through the NEI value and the parameter V defined by Vandesompele et al. [11], we obtain similar results, further supporting the method proposed in this report for assigning the optimal number of RGs to be considered for normalization. Interestingly, Figure 4 clearly demonstrates that the use of two or more RGs is a more efficient normalization method than using individual RGs, and this affirmation is more evident in the average NEI values of the less stable RGs. In this report, we propose the use of *miR-16*/*5S* and *B2M*/*18S* as the normalizers of microRNA and mRNA liver expression in rat models of acute hepatotoxicity. Each of the RGs in both pairs of genes belongs to differents biological classes and present different physiological functions in the liver.

Lastly, through the comparative evaluation of the analysis of putative RGs for both microRNA and mRNA normalization, it is clear that microRNA expression studies have certain advantages over mRNA in these models of acute hepatotoxicity. First, in the mRNA group, several genes were discarded because they exhibited altered expression between the control and the treated groups. The observation of the expression stability output values, i.e., SD, M or stability for each program is linked to this difference. It is also clear that candidate RGs for microRNA are more stably expressed than candidate RGs for mRNA expression studies. This difference is confirmed by the lower NEI values for the microRNA RGs compared to those of the mRNA RGs. These results confirm the importance of RG selection, especially in mRNA studies.

In conclusion, we present the first experimentally validated comparison of RGs for the normalization of microRNA and mRNA qPCR expression data in rat models of acute hepatotoxicity. The study was developed following an original workflow, where the confirmation of altered expression due to treatment is demonstrated to be a main issue in hepatotoxicity models. The combined use of *miR-16*/*5S* and *B2M*/*18S* were validated as the optimal normalization method for microRNA and mRNA expression data from liver, respectively, in rat models of acute hepatotoxicity. Therefore, the normalization methods proposed in this report will contribute to improve studies on the mechanism of hepatotoxicity of xenobiotics providing more reliable and accurate expression measurements.

## Materials and Methods

### Ethics Statement

All experiments with animals were performed according to the recommendations in the Guide for the Care and Use of Laboratory Animals of the School of Biochemical and Pharmaceutical Sciences, National University of Rosario, Argentina (Res. (CD) N° 267/020). The protocols were approved by the Animal Care and Use Committee of the School of Biochemical and Pharmaceutical Sciences, and included in a Research project accredited by the National University of Rosario, Argentina (BIO226, Res. (CS) N° 544/2009).

### Animals and chemicals employed

Adult male Wistar rats (300–350 g; School of Biochemical and Pharmaceutical Sciences, National University of Rosario) were used in the study. The animals were provided a standard diet and water *ad libitum* and housed in a temperature (21–23°C)- and humidity (45–50%)-controlled room under a constant 12 h light, 12 h dark cycle. All animals received humane care, according to the Guide for the Care and Use of Laboratory Animals (School of Biochemical and Pharmaceutical Sciences, National University of Rosario, Argentina) and the protocols were approved by the Animal Care and Use Committee of the School of Biochemical and Pharmaceutical Sciences. The hepatotoxins, AA, CT, GA and TA, were purchased from Sigma Chemical Co. (St. Louis, MO, USA).

#### Assessment of liver injury

Different animals received a unique intraperitoneal injection of vehicle and different doses of each hepatotoxin under study. The animals were sacrificed 24 h later. The dosages of each chemical compound administered were as follows: AA (0.3, 0.6 and 1.2 g/kg body weight; n = 5); CT (0.1, 0.4 and 1 ml/kg body weight; n = 5); GA (0.2, 0.5 and 0.9 g/kg body weight; n = 5) and TA (10, 50 and 150 mg/kg body weight; n = 5). The vehicles used for each xenobiotic were: 1% CMC (carboxymethyl cellulose, 10 ml/kg body weight, AA); corn oil (4.4 ml/kg body weight, CT) and saline solution (6 ml/kg body weight, GA and TA). Based on our dose-response curve analysis, we decided to study the selection of RGs in the livers of rats treated with the highest doses of each hepatotoxin and the livers of rats administered with each vehicle (n = 5).

At the end of each experiment, the rats were anesthetized and sacrificed by pneumothorax and the liver and blood samples were collected. The correct establishment of the acute hepatotoxicity models by each toxin was confirmed by the determination of plasma hepatotoxicity markers and histopathological examination. The plasma alanine aminotransferase and aspartate aminotransferase levels were assessed using commercial kits (Roche Diagnostics, GmbH, D- 68298, Mannheim, Germany) and a Roche-Hitachi Modular Autoanalyzer (Roche Diagnostics). The histological evaluation was performed using haematoxylin and eosin liver-stained sections and light microscopy.

#### RNA purification

The total RNA from the livers of the vehicle- and hepatotoxin-treated rats was isolated using the TRIzol reagent (Invitrogen, San Diego, CA, USA), according to the instructions of the manufacturer. Five pieces of different parts of the liver were used in each case. The homogenization of the samples was performed with a tissue-grinding tube and pestle, using 1 ml of TRIzol reagent per 10 mg of liver tissue. The RNA concentration and purity were determined measuring the absorbance at 260 and 280 nm using a NanoVue UV spectrophotometer (GE Healthcare, Piscataway, NJ, USA). The RNA integrity was assessed by the 18S and 28S band intensity ratio after 1.5% agarose gel electrophoresis visualized by ethidium bromide staining. The RNA was stored at −70°C for future use. Only those samples with a 260/280 ratio of approximately 2 (1.9 to 2.2) and a 28S/18S ratio ≥1.8 were used.

### Candidate reference genes and primer design

Four microRNAs (*miR-16*, *miR-103*, *miR-191* and *miR-Let7a*) and three small RNA genes (*5S*, *U6* and *RNU48*) were selected as candidate RGs for the normalization of the microRNA RT-qPCR data. Additionally, eight putative RGs were selected for the normalization of our mRNA RT-qPCR expression studies: seven mRNAs (*ALB*, *ACTB*, *B2M*, *CYCA*, *GAPDH*, *HPRT1* and *SDHA*) and one ribosomal RNA (*18S*). The primer sequences of the candidate RGs, with their corresponding bibliographic reference and amplicon sizes, are listed in Table 2. The NCBI (National Center for Biotechnology Information [http://www.ncbi.nlm.nih.gov]) and Ensembl (Ensembl Genome Browser [http://www.ensembl.org/index.html]) databases were used to search for available rat gene sequences to design primers using Primer3Plus [51], taking into account the possible secondary structures of the amplicon (Mfold) [52] and the amplicon specificity of the primers (Blast) [53]. The reaction conditions were optimized by determining the optimal annealing temperature and primer concentration.

### cDNA synthesis and real-time qPCR

The expression levels of microRNAs and small RNAs were measured by Stem-Loop RT-qPCR [31] and the expression levels of the mRNAs were determined by standard RT-qPCR [8], [10]. Prior to the reverse transcription reaction, 1 µg of the total RNA was treated with RQ1 RNase-free DNase (Promega, Madison, WI, USA) according to the protocol of the manufacturer. The first-strand cDNA synthesis was performed using SuperScript III Reverse Transcriptase (Invitrogen) according to the instructions of the manufacturer. For the cDNA synthesis of the microRNAs and small RNAs, the reaction mixture included Stem-Loop Oligos specific for each microRNA and specific primers for *U6* and *5S*. The cDNA synthesis of the mRNAs was performed using both a poly-dT primer and an *18S* specific primer (see Table 2 for the primer sequences). The reactions were incubated at 16°C for 30 min, 42°C for 30 min, 50°C for 60 min and 70°C for 15 min in the case of the microRNA and small RNA retrotranscription. The thermal protocol for mRNA was as follows: 50°C for 60 min and 70°C for 15 min. The cDNA samples were diluted 1/50 for the microRNA and small RNA determination or 1/20 for the mRNA and *18S* determination. The cDNA was stored at −20°C.

The PCR reactions were performed using an Mx3000P Real-Time Thermocycler (Stratagene, La Jolla, CA, USA) using SYBR Green I (Invitrogen) in a final volume of 20 µl. The reaction mixture consisted of 2 µl of 10× PCR Buffer, 1.2 µl of 50 mM MgCl_2_, 0.4 µl of 10 mM GeneAmp dNTP Mix (Applied Biosystems, Foster City, CA, USA), 0.8 µl of 10× SYBR Green I (Invitrogen), 0.1 µl of 5 U/µl Platinum Taq DNA Polymerase (Invitrogen), 4 µl of 2.5 µM primer mix (forward and reverse primers) and 5 µl of diluted cDNA. The PCR reactions were initiated with a 1 min incubation at 95°C, followed by 40 cycles of 95°C for 15 s, 60°C for 30 s and 72°C for 40 s. A melting curve was performed at the end of the PCR run over the range of 55–95°C, increasing the temperature stepwise by 0.5°C every 2 s. The baseline and Cq were automatically determined using MxPro version 4.10 (Stratagene). No template controls were included for each primer pair and each qPCR reaction was carried out in duplicate. Gene-specific amplification was confirmed by a single peak in the melting-curve analysis and a single band on a 2% agarose gel stained with ethidium bromide. The sample maximization method criterion was used to establish the run layout.

A dilution series was created with random cDNA from our sample group to construct standard curves for each primer pair. The qPCR reactions were performed, as described above, in duplicate. The mean Cq values for each serial dilution were plotted against the logarithm of the cDNA dilution factor. The amplification efficiency for each RG gene assay was calculated from the expression [10^(1/-S)^-1]×100%, where S represents the slope of the linear regression [8], [10].

### Data analysis

The Cq values were converted into RQ via the delta-Cq method [54], incorporating the calculated amplification efficiency for each primer pair [55]. The RQ values were calculated using the average Cq of all of the samples studied as the calibrator [13]. For all of the statistical analyses, the expression data were converted to logarithmic values to obtain symmetrical data. An unpaired one-tailed or two-tailed t-test was used to compare two separate sets of independent samples from the control and the treated rats. The one-way-ANOVA test was used when more than two groups were compared, followed by the Student-Newman–Keuls test for multiple comparison. In all cases, p values of less than 0.05 were considered statistically significant. Normality was assessed using the Shapiro-Wilk test. The statistical procedures were performed with the R program [http://www.R-project.org], v2.13.1.

Candidate RGs that presented statistically significant differences in their RQ values between the control and the treated groups were confirmed in a new set of animals that were treated using the same dosage (n = 5 each group). Lastly, the RQ differences between the control and the treated groups were normalized using the RGs that were proposed afterwards. The NRQ belonging to each group was obtained applying the normalization factor, which was calculated as the geometric mean of the relative expression of the RG selected.

To calculate the expression stability of the candidate RGs, the following software was used: BestKeeper version 1 [14] [http://gene-quantification.com/bestkeeper.html], geNorm version 3.5 [11] [http://medgen.ugent.be/~jvdesomp/genorm/] and NormFinder version 0953 [15] [http://www.mdl.dk/publicationsnormfinder.htm]. These freely available software packages are application tools for Microsoft Excel. The stability measurements produced by geNorm, NormFinder and BestKeeper were combined to establish a consensus rank of the genes applying the RankAggreg package [http://cran.rproject.org/web/packages/RankAggreg/index.html] of the R project [16]. The package RankAggreg has the ability to combine lists from different sources, which may not otherwise be directly comparable, as was performed in a previous work [50].

The simulated expression of a hypothetical gene of interest was used to assess the efficiency of a chosen normalization method (identity and number of genes selected). We used a modification of the original strategy reported by Ponton et al. [17]. Different normalization methods were evaluated, including a) individual testing of all the RGs one by one or b) a combination of RGs added stepwise according to the previously established stability ranking. These methods were evaluated in two hypothetical situations: with an increased and a decreased expression of the hypothetical gene of interest in the treated groups with respect to the control groups. The simulation of the expression of the hypothetical gene of interest was performed by assigning the same RQ value for all of the samples within each experimental group. For the control samples, this value was set to 1 in both of the hypothetical situations and the RQ values in the corresponding treated group were adjusted to the minimum (or maximum) value that produced a significant increase (or decrease) in the log-transformed NRQ between the groups using the t-test (p = 0.05). Lastly, we calculated the ratio between the average NRQ value of the gene of interest in the treated and the control groups, and this value was defined as the NEI for every regulation condition corresponding to a specific normalization method and hepatotoxin. Therefore, in both cases, the NEI represents the x-fold up (or down) change that reflects the minimum expression difference that can be detected in the studied condition. The lower is the NEI value, the higher is the efficiency of the normalization method applied. Considering that the NEI is the NRQ value of a hypothetical gene under specific conditions, we can estimate the error associated with this determination through the error propagation method reported by Hellemans et al. [13]. In this report, the average NEI value (considering all of the hepatotoxins) for situations of up- and down-regulation is used to evaluate each normalization method and to establish the optimal number of RGs to be used. A detailed description of the arithmetic procedure through an example is offered in Data S3.

## Supporting Information

Figure S1 **Representative amplification and melt-curve profiles of Real-Time qPCR assays.**
(PDF)

Figure S2 **Experimental validation of all expression data of ribosomal genes evaluating their inclusion within the dynamic range of RT-qPCR.**
(PDF)

Figure S3 **Study of normalization efficiency of candidate reference genes for microRNA and mRNA in differents models of hepatotoxicity.**
(PDF)

Table S1 **Checklist MIQE.**
(PDF)

Table S2 **Normality assessment of log-transformed expression data of candidate reference genes by Shapiro-Wilk test.**
(PDF)

Data S1 **qPCR raw data in RDML language.**
(RAR)

Data S2 **Outputs of geNorm, NormFinder, BestKeeper and RankAggreg.**
(PDF)

Data S3 **Example of calculation of the Normalization efficiency index (NEI) for different normalization methods in an study of liver gene expression between control and carbon tetrachloride (CT) treated rats.**
(XLS)
