# Supplementary material for: Reference Genes for Real-Time PCR Quantification of MicroRNAs and Messenger RNAs in Rat Models of Hepatotoxicity
Source: PLoS One. 2012 May 1;7(5):e36323. doi: 10.1371/journal.pone.0036323 (PMC3341372; doi:10.1371/journal.pone.0036323)

Figure S1

Representative amplification and melt-curve profiles of Real-Time qPCR assays

Candidate reference genes for microRNA normalization

Candidate reference genes for mRNA normalization

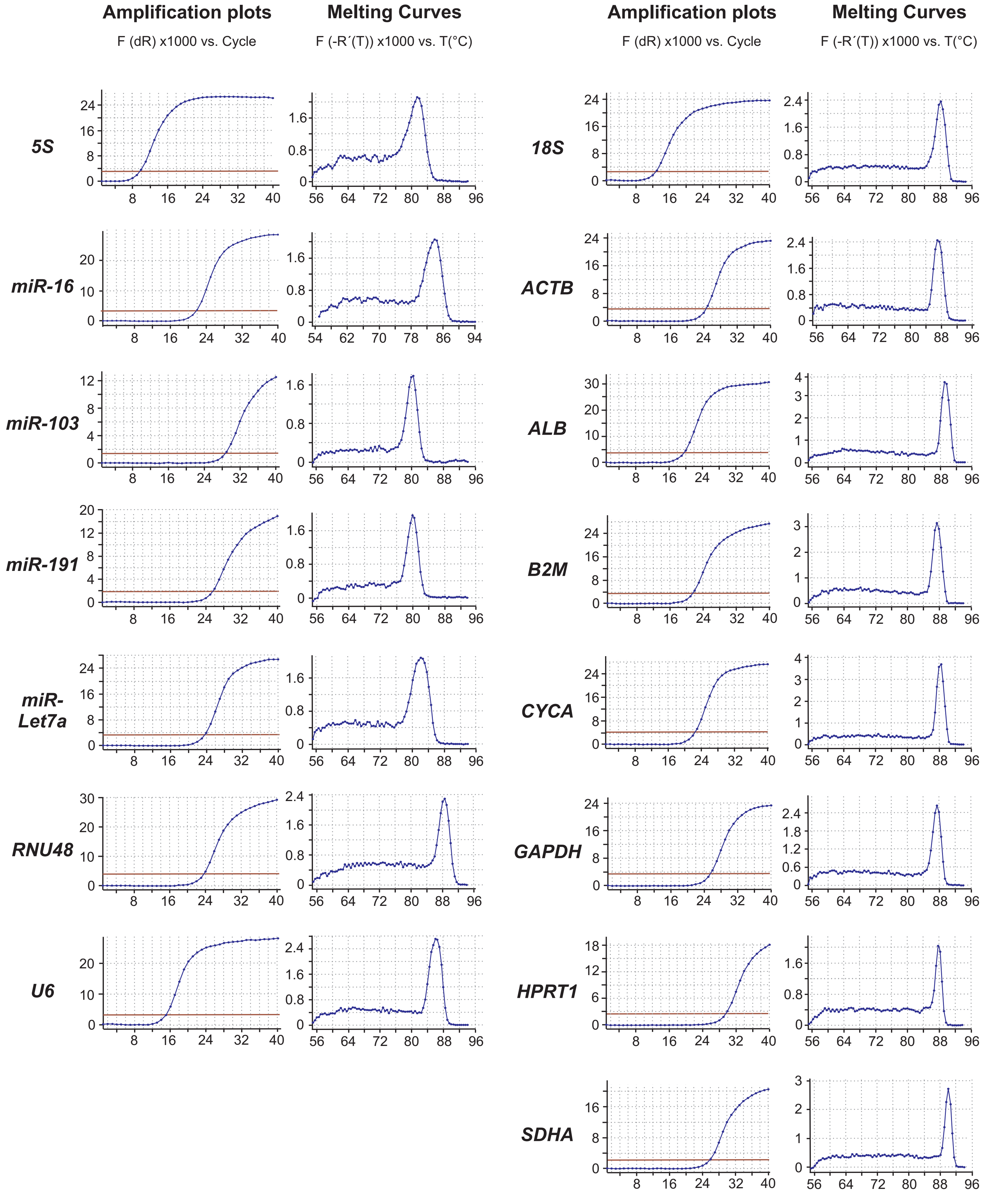

Representative amplification and melt-curve profiles of Real-Time qPCR assays

Genes of interest

Amplification plots | **Melting Curves** | **Amplification plots** | **Melting Curves**

F (dR) x1000 vs. Cycle | F (-R´(T)) x1000 vs. T(°C) | F (dR) x1000 vs. Cycle | F (-R´(T)) x1000 vs. T(°C)

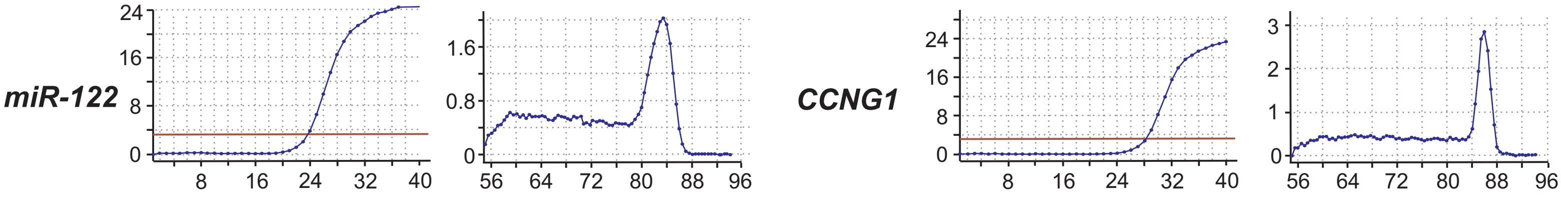

Supplement: Figure S1 — Representative amplification and melt-curve profiles of Real-Time qPCR assays. (PDF) [file pone.0036323.s001.pdf]
