# Supplementary material for: Reference Genes for Real-Time PCR Quantification of MicroRNAs and Messenger RNAs in Rat Models of Hepatotoxicity
Source: PLoS One. 2012 May 1;7(5):e36323. doi: 10.1371/journal.pone.0036323 (PMC3341372; doi:10.1371/journal.pone.0036323)
Supplement: Figure S2 — Experimental validation of all expression data of ribosomal genes evaluating their inclusion within the dynamic range of RT-qPCR. (PDF) [file pone.0036323.s002.pdf]

Figure S2

Experimental validation of all expression data of ribosomal genes evaluating their inclusion within the dynamic range of RT-qPCR

Studies of the dynamic range of qPCR amplification reaction

5S Ribosomal RNA

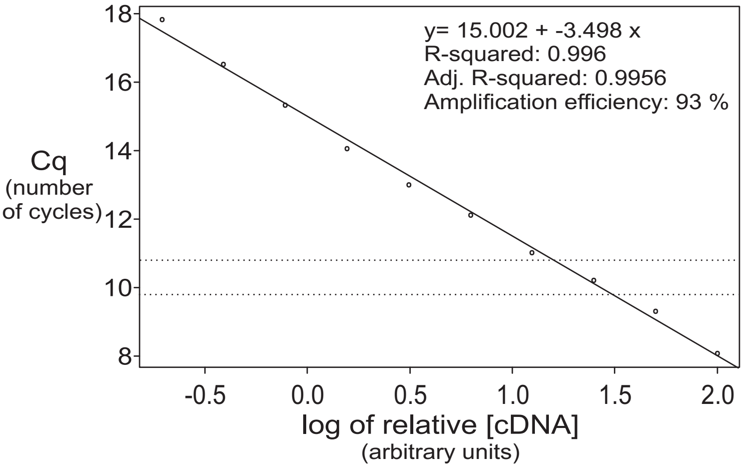

18S Ribosomal RNA

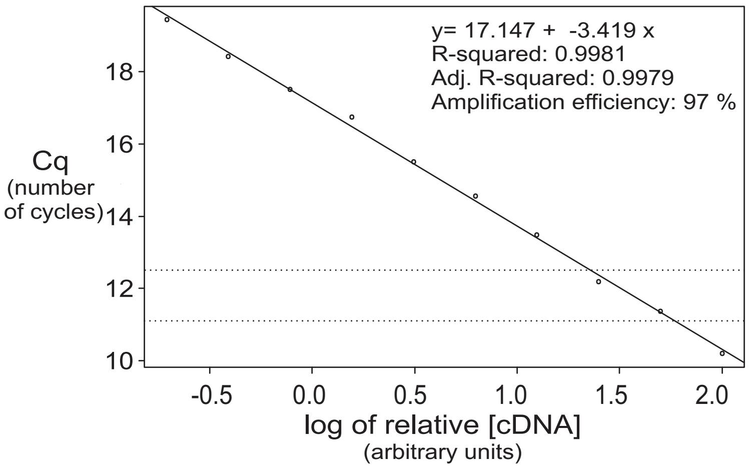

Studies of the dynamic range of reverse transcription reaction

5S Ribosomal RNA

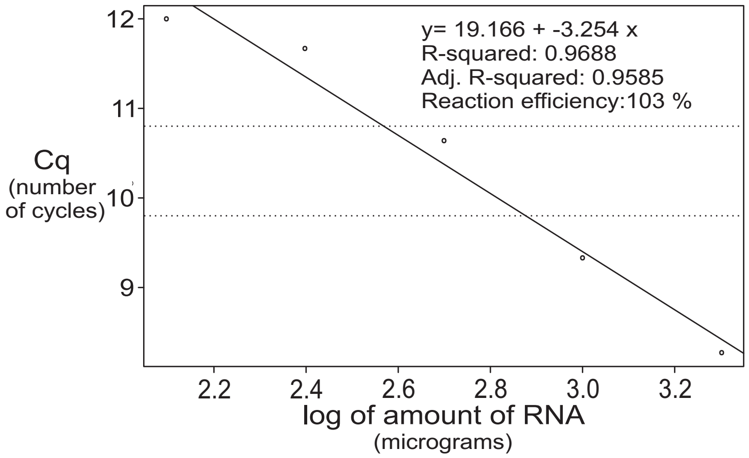

Transcript measurement of ribosomal genes in serial cDNA dilution samples by real-time PCR (upper panel) and in serial dilution of RNA samples by RT-qPCR (lower panel) are shown. The linear regression in the plots of quantification cycle (Cq) against the Log of the relative concentration or amount of starting material define the dynamic range. The linear regression was applied in all cases obtaining the correlation coefficients (R-squared and Adjusted R-squared) and their corresponding equation. The amplification/reaction efficiency of the PCR reaction was calculated from the expression [10(1/-S)-1] × 100 %, where S represents the slope of the linear regression. The Cq range of values of the expression data of the ribosomal genes from all the experiments of this report are shown in dotted lines.
