# Supplementary material for: Reference Genes for Real-Time PCR Quantification of MicroRNAs and Messenger RNAs in Rat Models of Hepatotoxicity
Source: PLoS One. 2012 May 1;7(5):e36323. doi: 10.1371/journal.pone.0036323 (PMC3341372; doi:10.1371/journal.pone.0036323)
Supplement: Figure S3 — Study of normalization efficiency of candidate reference genes for microRNA and mRNA in differents models of hepatotoxicity. (PDF) [file pone.0036323.s003.pdf]

Figure S3

Study of normalization efficiency of candidate reference genes for microRNA and mRNA.

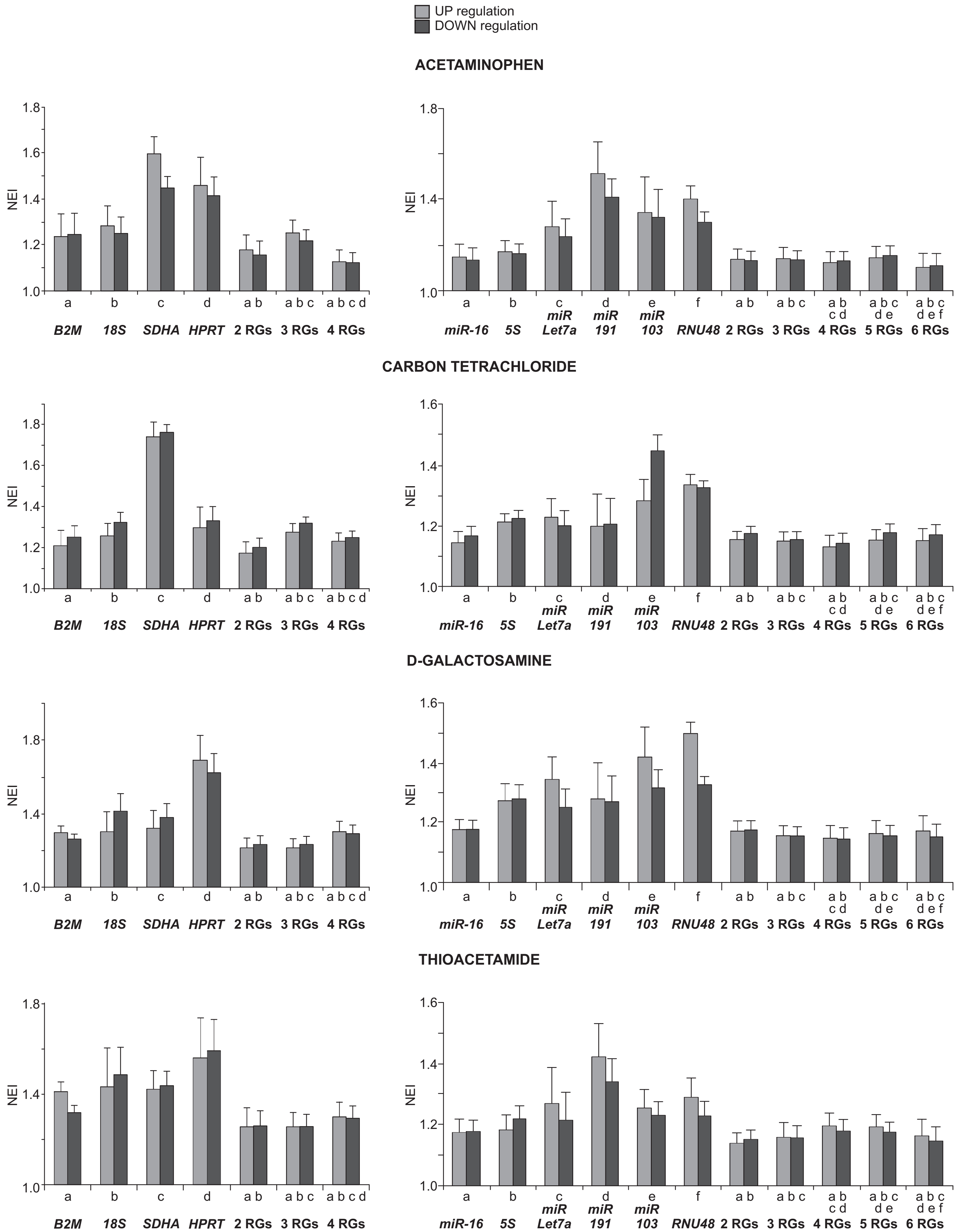

The figure shows the normalization efficiency index (NEI) calculated for each hepatotoxin in up- and down-regulation situations. We defined the NEI value as the minimum fold up- or down-regulation to observe a significant expression difference (t-test, p=0.05, n=5) between control and treated groups using a particular normalization method. The NEI is calculated by normalizing against individual reference genes or a combination of two or more reference gene added stepwise, following the left-to-right stability rank established previously. The error bars were determined through calculating the error propagation of the normalized relative quantity of transcript.
