# Supplementary material for: Reference Genes for Real-Time PCR Quantification of MicroRNAs and Messenger RNAs in Rat Models of Hepatotoxicity
Source: PLoS One. 2012 May 1;7(5):e36323. doi: 10.1371/journal.pone.0036323 (PMC3341372; doi:10.1371/journal.pone.0036323)
Supplement: Table S1 — Checklist MIQE. (PDF) [file pone.0036323.s004.pdf]

Table S1

Checklist MIQE

Item to check	Importance	
**Experimental design**		
Definition of experimental and control groups	E	**Experimental groups**: rats treated with hepatotoxins (acetaminophen, carbon tetrachloride, D-galactosamine, thioacetamide) at different dosis. **Control groups**: vehicle-treated rats
Number within each group	E	n=5 for dosis-response curves; n=5 for RGs evaluation in highest dosis-treated animales
Assay carried out by the core or investigator's laboratory?	D	
Acknowledgment of authors' contributions	D	
**Sample**		
Description	E	**Experimental samples**: livers from rats treated with hepatotoxics at different dosis. **Control samples**: livers from vehicle-treated rats
Volume/mass of sample processed	D	10 mg
Microdissection or macrodissection	E	Macrodissection
Processing procedure	E	Rats were anesthetized with intraperitoneal injection of ketamine (100 mg/kg rat) / xilazine (3 mg/kg rat) and sacrificed by induction of pneumotorax. Liver was isolated and washed with saline solution.
If frozen, how and how quickly?	E	Liver tissues were frozen in liquid N2 inmediatly after they were obtained and washed.
If fixed, with what and how quickly?	E	Not fixed
Sample storage conditions and duration	E	Samples were held at -70°C for up to one week before RNA isolation
**Nucleic acid extraction**		
Procedure and/or instrumentation	E	Total RNA was extracted using the TRIzol Reagent (Invitrogen) following manufacture's protocol. Homogenization of samples was performed in tissue grind tube and pestle using 1 mL of TRIzol Reagent per 10 mg of tissue (left liver lobe). Following homogenization, insoluble material from the homogenate was removed by centrifugation at 12,000 g for 10 minutes at 4°C. The purified RNA was dissolved in 40 ul DEPC-Treated Water and stored at -70°C.
Name of kit and details of any modifications	E	TRIzol Reagent (Invitrogen). We exactly followed manufacture's protocol.
Source of additional reagents used	D	Chloroform (Merck); 2-propanol (Merck); Ethanol (Merck); DEPC-Treated Water (Ambion)
Details of DNase or RNase treatment	E	1 ug of RNA was treated with 1 U of RQ1 RNase-Free DNase (Promega) in a 20 ul final volume reaction. Digestion of DNA was achieved with 30 minutes incubation at 37°C. The reaction was stopped with 1 ul of DNase Stop Solution (Promega) following 10 min incubation at 65°C for inactivation.
Contamination assessment (DNA or RNA)	E	reverse transcription controls (without enzyme) were performed in order to assess the absence of DNA in the RNA sample. For that purpose, RNA was processed as a normal sample in the RT step, except that no reverse transcriptase was added to the reaction mixture (see "complete reaction conditions" in Reverse Transcription).
Nucleic acid quantification	E	RNA concentration was determined by measuring the absorbance at 260 nm UV light
Instrument and method	E	NanoVue (GE Healthcare) UV spectrophotometer
Purity (A260/A280)	D	RNA purity was determined by measuring the absorbance ratio 260/280
Yeld	D	RNA yeld was calculated as the amount of RNA obtained (ug) per mg of tissue processed. The average yeld was 2.45, with a minimum value of 1.3 and a maximum value of 4.43.
RNA integrity: method/instrument	E	The RNA integrity was assessed by the 18S and 28S band intensity ratio after 1.5% agarose gel electrophoresis visualized by ethidium bromide staining. Only those samples with a 28S/18S ratio ≥1.7 were used.
RIN/RQI or Cq of 3' and 5' transcripts	E	Not applicable
Electrophoresis traces	D	
Inhibition testing (Cq dilutions, spike, or other)	E	The standard curve has been considered sufficient to rule out the presence of inhibitors of reverse-transcription activity or PCR, also taking into account the high quality of starting RNAs.

Reverse transcription		
Complete reaction conditions	E	REVERSE TRANSCRIPTION OF microRNAs AND SMALL RNAs: 10 ul of RNA (from the DNase treatment reaction mixture) was firstly incubated with 1 ul dNTP Mix 10 mM and 2 ul of a specific primers mixture, in a final volume of 13.5 ul. This mixture was heated to 65°C for 5 min and then incubated on ice for at least 1 min for primer annealing. Finally, the sample was mixed with 4 ul 5X First-Strand Buffer, 1 ul 0.1 M DTT, 1 ul RNase OUT 40 U/ul (Invitrogen) and 0.5 ul SuperScript III 200 U/ul (Invitrogen). Reactions were incubated in a GeneAmp PCR System 2400 (Perkin Elmer) at 16 °C for 30 min, 42 °C for 30 min, 50 °C for 60 min and 70°C for 15 min to stop the reaction. The 10 ul remaining of the DNase treatment reaction mixture was used for the No-RT control.
		REVERSE TRANSCRIPTION OF mRNA: 10 ul of RNA (from the DNase treatment reaction mixture) was firstly incubated with 0.5 ul Oligo dT 0.5 ug/ul, 1 ul dNTP Mix 10 mM and 2 ul of specific primer for 18S. This mixture was heated to 65°C for 5 min and then incubated on ice for at least 1 min for primer annealing. Finally, the sample was mixed with 4 ul 5X First-Strand Buffer, 1 ul 0.1 M DTT, 1 ul RNase OUT 40 U/ul (Invitrogen) and 0.5 ul SuperScript III 200 U/ul (Invitrogen). Reactions were incubated in a GeneAmp PCR System 2400 (Perkin Elmer) at 50 °C for 60 min and 70°C for 15 min to stop the reaction. The 10 ul remaining of the DNase treatment reaction mixture was used for the No-RT control.
Amount of RNA and reaction volume	E	Amount of RNA: 500 ng; Reaction volume: 20 ul
Priming oligonucleotide (if using GSP) and concentration	E	18S: 2.5 uM; U6: 0.75 uM; 0.75 uM 5S; 50 nM Stem Loop-Oligos for miR-16, miR-Let7a, RNU48, miR-191, miR-103, miR-122
Reverse transcriptase and concentration	E	SuperScript III (Invitrogen) in final concentration: 5 U/ul
Temperature and time	E	Specified in "Complete reaction conditions"
Manufacturer of reagents and catalogue numbers	D	DNase (Promega, Cat. M610A); GeneAmp dNTP Mix with dTTP (Applied Biosystem, Cat. N8080260); SuperScript III (Invitrogen, Cat. 18080-044); RNase Out (Invitrogen, Cat 10777-019)
Cqs with and without reverse transcription	D	
Storage conditions of cDNA	D	-20°C
**qPCR protocol**		
Complete reaction conditions	E	PCR reactions were performed in a Mx3000P Real Time Thermocycler (Stratagene) using SYBR Green I (Invitrogen) in final volume of 20 ul. Reaction mix consisted of 2 ul 10X PCR Buffer, 1.2 ul 50 mM MgCl2, 0.4 ul 10 mM GeneAmp dNTP Mix (Applied Biosystems), 0.8 ul SYBR Green I (Invitrogen), 0.1 ul 5 U/ul Platinum® Taq DNA Polymerase (Invitrogen), 4 ul 2.5 uM primer mix (forward and reverse primers) and 5 ul of 1/20 or 1/50 cDNA. The PCR reactions were initiated with 1 minute incubation at 95°C, followed by 40 cycles of 95°C for 15 seconds, 60°C for 30 seconds and 72°C for 40 seconds. All reactions were performed in duplicate.
Reaction volume and amount of cDNA/DNA	E	Reaction volume: 20 ul; amount of cDNA: 5 ul of 1/50 dilution for microRNA and smal RNA determination or 1/20 dilution for mRNA or rRNA determinations
Primer, (probe), Mg2, and dNTP concentrations	E	500 nM Primers; 3 mM MgCl2; 0.2 mM dNTP
Polymerase identity and concentration	E	Taq DNA Polymerase (Invitrogen, Cat. 10966-026) in final concentration: 0.025 U/ul
Buffer/kit identity and manufacturer	E	10X PCR Buffer, Minus Mg (Invitrogen)
Exact chemical composition of the buffer	D	
Additives (SYBR Green I, DMSO, and so forth)	E	SYBR® Green I Nucleic Acid Gel Stain (Invitrogen)
Manufacturer of plates/tubes and catalog number	D	PCR Strip Tubes (PCR-0208-C) and PCR Strip Caps (PCR-2CP-RT-C), both provided by Axygen
Complete thermocycling parameters	E	Initial denaturation: 95°C for 1 minute, then 40 cycles at 95°C for 15 seconds, 60°C for 30 seconds and 72°C for 40 seconds
Reaction setup (manual/robotic)	D	Manual
Manufacturer of qPCR instrument	D	Mx3000P Real Time Thermocycler (Stratagene)

qPCR validation		
Evidence of optimization (from gradients)	D	
Specificity (gel, sequence, melt, or digest)	E	Melting curve analysis, ramping from 55°C to 95°C in 23 minutes, where fluorescence data are measured continously (measured melting temperature values are provided as Supplementary data). Gene-specific amplification was confirmed by a single band in 2% agarose gel electrophoresis stained with ethidium bromide. No template controls (no cDNA in PCR) were run for each gene to detect unspecific amplification and primer dimerization.
For SYBR Green I, Cq of the NTC	E	The signal of the amplification plot was very late (Cq>34) and therefore there was a high Cq value difference between the negative control and all the cDNA sample. Except for NTC of 18S, which resulted in Cq = 23, but with a large difference in Cq between negative control and cDNA samples (9 cycles).
Calibration curves with slope and y intercept	E	**18S**: y = -3.4565x + 18.583; **ALB**: y = -3.7177x + 23.337; **CYCA**: y = -3.4558x + 29.735; **B2M**: y = -3.6546x + 28.884; **HPRT**: y = -3.7614x + 36.36; **ACTB**: y = -3.1409x + 34.767; **GAPDH**: y = -3.6999x + 33.184; **SDHA:** -3.4508x + 32.495; **5S**: y = -3.6192x + 18.903; **miR-16**: y = -3.87x + 31.098; **miR-Let7a**: y = -3.5412x + 33.203; **RNU48**: y = -3.7538x + 31.008; **U6**: y = -3.5078 + 18.347; **miR-191**: -3.2389x + 31.87; **miR-103**: -3.2621x + 35.334, **miR-122**: -3.4731x + 31.654, **CCNG1**: -3.8298x + 34.697
PCR efficiency calculated from slope	E	**18S**: 97.3%; **ALB**: 92.9%; **CYCA**: 97.4%; **B2M**: 93.9%; **HPRT**: 92.0%; **ACTB**: 104.0%; **GAPDH**: 93.2%; **SDHA**: 97.5%; **5S**: 94.5%; **miR-16**: 90.7%; **miR-Let7a**: 95.8%; **RNU48**: 92.3%; **U6**: 96.5%; **miR-191**: 103.9 %; **miR-103**: 103.7 %; **miR-122**: 97 %; **CCNG1**: 92 %
CIs for PCR efficiency or SE	D	
r2 of calibration curve	E	**18S**: 0.9961; **ALB**: 0.9968; **CYCA**: 0.9830; **B2M**: 0.9955; **HPRT**: 0.9924; **ACTB**: 0.9951; **GAPDH**:0.9937; **SDHA**: 0.9935; **5S**: 1.0000; **miR-16**: 0.9976; **miR-Let7a**: 0.9774; **RNU48**: 0.998; **U6**: 0.998; **miR-191**: 0.997; **miR-103**: 0.991; **miR-122**: 0.9905; **CCNG1**: 0.9868
Linear dynamic range	E	In average, linear dynamic range was considered taking into account the linearity of the standard curves; from 1/10 dilution of cDNA to 1/800 dilution
Cq variation at LOD	E	
CIs throughout range	D	
Evidence for LOD	E	
If multiplex, efficiency and LOD of each assay	E	Not applicable

**Data analysis**		
qPCR analysis program (source, version)	E	MxPro QPCR Software
Method of Cq determination	E	The threshold is determined using the Amplification-based Threshold method. The threshold is used to specify Cq values of samples.
Outlier identification and disposition	E	None of the Cq values was discarded
Results for NTCs	E	The signal of the amplification plot was very late (Cq>34) and therefore there was a high Cq value difference between the negative control and all the cDNA sample. Except for NTC of 18S, which resulted in Cq = 23, but with a large difference in Cq between negative control and cDNA samples (9 cycles).
Justification of number and choice of reference genes	E	This is a study for the selection of reference genes
Description of normalization method	E	Described in text
Number and concordance of biological replicates	D	
Number and stage (RT or qPCR) of technical replicates	E	qPCR reactions were performed in duplicate
Repeatability (intraassay variation)	E	Mean standard deviation of duplicates: 0.10
Reproducibility (interassay variation, CV)	D	
Power analysis	D	
Statistical methods for results significance	E	Student´s t Test
Software (source, version)	E	R Project
Cq or raw data submission with RDML	D	In Supplementary data
**qPCR target information**		
Gene symbol	E	Table 1 in paper
Sequence accession number	E	Table 1 in paper
Location of amplicon	D	
Amplicon length	E	Table 1 in paper
In silico specificity screen (BLAST, and so on)	E	Described in text
Pseudogenes, retropseudogenes, or other homologs?	D	
Sequence alignment	D	
Secondary structure analysis of amplicon	D	
Location of each primer by exon or intron (if applicable)	E	For microRNA and smal RNA this is not applicable; for the rest of genes, see references cited in Table 1
What splice variants are targeted?	E	
**qPCR oligonucleotides**		
Primer sequences	E	Table 1 in paper
RTPrimerDB identification number	D	
Probe sequences	D	
Location and identity of any modifications	E	No modifications were done
Manufacturer of oligonucleotides	D	Alpha DNA. IDT oligo
Purification method	D	Desalted

qPCR target information		
Gene symbol	E	Table 1 in paper
Sequence accession number	E	Table 1 in paper
Location of amplicon	D	
Amplicon length	E	Table 1 in paper
In silico specificity screen (BLAST, and so on)	E	Described in text
Pseudogenes, retropseudogenes, or other homologs?	D	
Sequence alignment	D	
Secondary structure analysis of amplicon	D	
Location of each primer by exon or intron (if applicable)	E	
What splice variants are targeted?	E	
**qPCR oligonucleotides**		
**Primer sequences**	E	Table 1 in paper
**RTPrimerDB identification number**	D	-
**Probe sequences**	D	-
**Location and identity of any modifications**	E	No modifications were done
**Manufacturer of oligonucleotides**	D	Alpha DNA, IDT oligo
**Purification method**	D	Desalted
