# Supplementary material for: Reference Genes for Real-Time PCR Quantification of MicroRNAs and Messenger RNAs in Rat Models of Hepatotoxicity
Source: PLoS One. 2012 May 1;7(5):e36323. doi: 10.1371/journal.pone.0036323 (PMC3341372; doi:10.1371/journal.pone.0036323)
Supplement: Table S2 — Normality assessment of log-transformed expression data of candidate reference genes by Shapiro-Wilk test. (PDF) [file pone.0036323.s005.pdf]

Table S2

Normality assessment of log-transformed expression data of candidate reference genes by Shapiro-Wilk test

Gen Symbol	W	p-value
**Candidate reference genes for microRNA normalization**		
5S	0.9494	0.1088
miR-16	0.9683	0.3985
miR-103	0.9839	0.8769
miR-191	0.9771	0.6642
miR-Let7a	0.9576	0.193
RNU48	0.9634	0.2877
**Candidate reference genes for mRNA normalization**		
18S	0.9817	0.814
B2M	0.9898	0.9825
HPRT1	0.967	0.3672
SDHA	0.9817	0.8118

W: the value of the Shapiro-Wilk statistic, p.value: an approximate p-value for the test.
