# Supplementary material for: Reference Genes for Real-Time PCR Quantification of MicroRNAs and Messenger RNAs in Rat Models of Hepatotoxicity
Source: PLoS One. 2012 May 1;7(5):e36323. doi: 10.1371/journal.pone.0036323 (PMC3341372; doi:10.1371/journal.pone.0036323)
Supplement: Data S2 — Outputs of geNorm, NormFinder, BestKeeper and RankAggreg. (PDF) [file pone.0036323.s007.pdf]

Data S2

Candidate reference genes for microRNA normalization

BestKeeper version 1

http://gene-quantification.com/bestkeeper.html

Result - Genes: Repeated Pair-wise Correlation Analysis & Regression Analysis of BestKeeper vs. Housekeeping Genes

CP data of housekeeping Genes:

	5S	mir16	mir 103	mir 191	mir let7a	RNU48
	HKG 1	HKG 2	HKG 3	HKG 4	HKG 5	HKG 6
n	35	35	35	35	35	35
geo Mean [CP]	10.33	22.80	30.09	26.45	24.10	23.46
ar Mean [CP]	10.33	22.80	30.10	26.45	24.10	23.46
min [CP]	9.82	22.27	29.00	25.33	23.42	22.48
max [CP]	10.78	23.44	31.06	27.57	24.96	24.68
std dev [± CP]	0.19	0.25	0.39	0.35	0.25	0.35
CV [% CP]	1.82	1.08	1.30	1.34	1.03	1.50
min [x-fold]	-1.39	-1.37	-2.22	-2.27	-1.56	-1.83
max [x-fold]	1.33	1.46	2.02	2.27	1.75	2.12
std dev [± x-fold]	1.13	1.17	1.28	1.25	1.17	1.25

Pearson correlation coefficient (r)

vs.	HKG 1	HKG 2	HKG 3	HKG 4	HKG 5	HKG 6
HKG 2	0.415	-	-	-	-	-
p-value	0.013	-	-	-	-	-
HKG 3	-0.045	0.182	-	-	-	-
p-value	0.796	0.297	-	-	-	-
HKG 4	0.149	0.351	0.715	-	-	-
p-value	0.391	0.039	0.001	-	-	-
HKG 5	0.061	0.072	0.176	-0.021	-	-
p-value	0.729	0.684	0.311	0.905	-	-
HKG 6	-0.020	0.057	-0.222	-0.225	0.440	-
p-value	0.913	0.743	0.199	0.193	0.008	-
BestKeeper vs.	HKG 1	HKG 2	HKG 3	HKG 4	HKG 5	HKG 6
coeff. of corr. [r]	0.589	0.638	0.515	0.606	0.483	0.326
p-value	0.001	0.001	0.002	0.001	0.003	0.056

Regression Analysis: HKG vs. BestKeeper

	5S	mir16	mir 103	mir 191	mir let7a	RNU48
	HKG 1	HKG 2	HKG 3	HKG 4	HKG 5	HKG 6
	vs.	vs.	vs.	vs.	vs.	vs.
	BK	BK	BK	BK	BK	BK
coeff. of corr. [r]	0.59	0.64	0.52	0.61	0.48	0.33
coeff. of det. [r^2]	0.35	0.41	0.27	0.37	0.23	0.11
intercept [CP]	-5.72	1.01	2.80	-5.22	7.50	7.05
slope [CP]	0.74	1.00	1.25	1.46	0.76	0.75
SE [CP]	±0.202	±0.241	±0.416	±0.38	±0.275	±0.436
p-value	0.001	0.001	0.002	0.001	0.003	0.056
Power of HKG [x-fold]	1.60	1.81	2.50	2.90	1.64	1.59

Candidate reference genes for microRNA normalization

geNorm™ version 3.5

http://medgen.ugent.be/~jvdesomp/genorm/

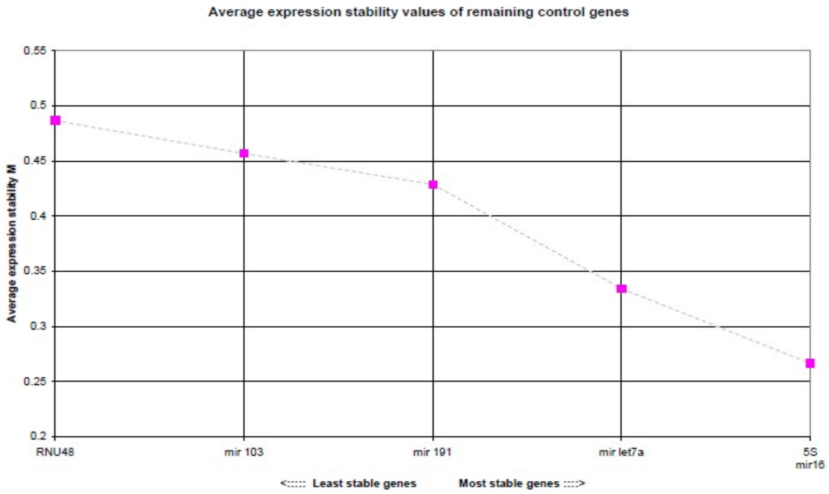

Candidate reference genes for microRNA normalization

NormFinder version 0953

http://www.mdl.dk/publicationsnormfinder.htm

Gene name	Stability value
5S	0.152
mir16	0.138
mir 103	0.249
mir 191	0.206
mir let7a	0.162
RNU48	0.253

**Best gene Stability value**	mir16 0.138
**Best combination of two genes**	5S and mir 191
**Stability value for best combination of**	0.110

Intragroup variation

Group identifier	1	2	3	4	5	6	7
5S	0.001	0.006	0.006	0.016	0.044	0.115	0.014
mir16	0.001	0.000	0.001	0.002	0.010	0.008	0.003
mir 103	0.023	0.085	0.083	0.005	0.008	0.067	0.025
mir 191	0.038	0.182	0.003	0.026	0.025	0.060	0.063
mir let7a	0.003	0.091	0.031	0.047	0.002	0.018	0.018
RNU48	0.059	0.151	0.054	0.057	0.034	0.108	0.083

Intergroup variation

Group identifier	1	2	3	4	5	6	7
5S	0.135	-0.038	0.006	0.096	-0.077	0.047	-0.168
mir16	-0.004	-0.209	0.067	0.094	0.037	0.181	-0.167
mir 103	-0.094	-0.036	-0.193	-0.259	0.255	-0.007	0.334
mir 191	-0.310	-0.099	0.033	0.001	0.082	-0.016	0.310
mir let7a	0.143	0.172	-0.002	-0.061	-0.066	-0.061	-0.125
RNU48	0.130	0.210	0.089	0.130	-0.230	-0.145	-0.183

Candidate reference genes for microRNA normalization

RankAggreg package of R project

http://cran.rproject.org/web/packages/RankAggreg/index.html

Rank aggregation without weights

```
x <- matrix(c
("2", "4", "1", "3", "5", "6",
"2", "1", "5", "4", "3", "6",
"2", "1", "5", "4", "3", "6")
byrow=TRUE, ncol=6)
> (toplist <- BruteAggreg(x, 6))
```

The optimal list is:
2 1 5 4 3 6

Algorithm: BruteForce
Distance: Spearman
Score: 2

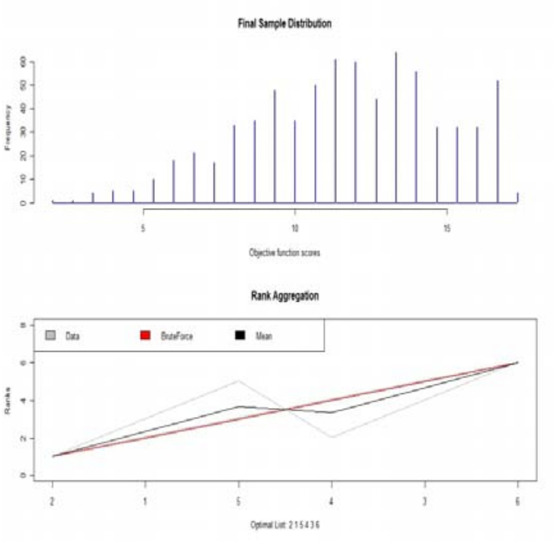

Rank aggregation without weights

```
x <- matrix(c
("2", "4", "1", "3", "5", "6",
"1", "2", "5", "4", "3", "6",
"2", "1", "5", "4", "3", "6")
byrow=TRUE, ncol=6)
> (toplist <- BruteAggreg(x, 6))
```

The optimal list is:
2 1 5 4 3 6

Algorithm: BruteForce
Distance: Spearman
Score: 2.666667

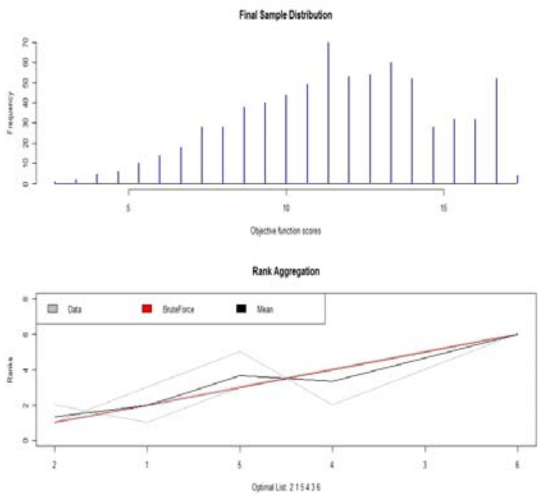

Gene references

1	*5S*
2	*miR-16*
3	*miR-103*
4	*miR-191*
5	*miR-Let7a*
6	*RNU48*

Candidate reference genes for mRNA normalization

BestKeeper version 1

http://gene-quantification.com/bestkeeper.html

Result - Genes: Repeated Pair-wise Correlation Analysis & Regression

Analysis of BestKeeper vs. Housekeeping Genes

CP data of housekeeping Genes:

	18S	B2M	HPRT	SDHA
	HKG 1	HKG 2	HKG 3	HKG 4
n	35	35	35	35
geo Mean [CP]	11.77	22.09	29.34	26.32
ar Mean [CP]	11.78	22.09	29.35	26.33
min [CP]	11.09	21.20	28.37	25.11
max [CP]	12.45	22.87	30.93	27.41
std dev [± CP]	0.28	0.31	0.47	0.48
CV [% CP]	2.39	1.42	1.61	1.83
min [x-fold]	-1.71	-1.75	-1.81	-2.24
max [x-fold]	1.71	1.64	2.63	2.06
std dev [± x-fold]	1.25	1.28	1.45	1.46

Pearson correlation coefficient (r)

vs.	HKG 1	HKG 2	HKG 3	HKG 4
HKG 2	-0.048	-	-	-
p-value	0.781	-	-	-
HKG 3	-0.238	0.287	-	-
p-value	0.168	0.095	-	-
HKG 4	0.230	0.220	0.100	-
p-value	0.183	0.203	0.566	-

BestKeeper vs.	HKG 1	HKG 2	HKG 3	HKG 4
coeff. of corr. [r]	0.587	0.540	0.401	0.686
p-value	0.001	0.001	0.017	0.001

Regression Analysis: HKG vs. BestKeeper

	18S	B2M	HPRT	SDHA
	HKG 1	HKG 2	HKG 3	HKG 4
	vs.	vs.	vs.	vs.
	BK	BK	BK	BK
coeff. of corr. [r]	0.59	0.54	0.40	0.69
coeff. of det. [r^2]	0.35	0.29	0.16	0.47
intercept [CP]	-4.79	4.90	10.79	-4.91
slope [CP]	0.78	0.81	0.88	1.48
SE [CP]	±0.29	±0.34	±0.538	±0.42
p-value	0.001	0.001	0.017	0.001
Power of HKG [x-fold]	1.85	1.67	1.71	2.68

Candidate reference genes for mRNA normalization

geNorm™ version 3.5 Output

http://medgen.ugent.be/~jvdesomp/genorm/

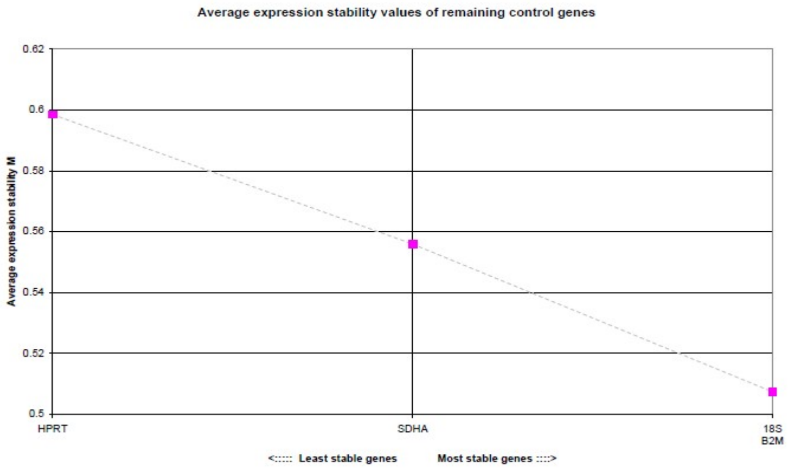

Candidate reference genes for mRNA normalization

NormFinder version 0953

http://www.mdl.dk/publicationsnormfinder.htm

Gene name	Stability value		
18S	0.180	**Best gene Stability value**	B2M 0.172
B2M	0.172		
HPRT1	0.230	**Best combination of two genes**	B2M and SDHA
SDHA	0.209	**Stability value for best combination of two genes**	0.128

Intragroup variation

Group identifier	1	2	3	4	5	6	7
18S	0.039	0.005	0.061	0.026	0.191	0.063	0.238
B2M	0.017	0.005	0.001	0.005	0.011	0.016	0.002
HPRT1	0.090	0.270	0.121	0.008	0.090	0.097	0.047
SDHA	0.084	0.161	0.137	0.231	0.008	0.008	0.026

Intergroup variation

Group identifier	1	2	3	4	5	6	7
18S	0.028	-0.063	-0.010	-0.031	0.045	0.174	-0.144
B2M	-0.165	-0.223	-0.069	0.070	0.035	0.091	0.262
HPRT1	-0.029	0.256	-0.127	0.143	-0.117	-0.237	0.109
SDHA	0.166	0.030	0.205	-0.183	0.038	-0.028	-0.228

Candidate reference genes for mRNA normalization

RankAggreg package of R project

http://cran.rproject.org/web/packages/RankAggreg/index.html

Rank aggregation without weights

x <- matrix(c
("4", "1", "2", "3"),
"2", "1", "4", "3",
+ "2", "1", "4", "3")
byrow=TRUE, ncol=4)
(toplist <- BruteAggreg(x, 4))

The optimal list is:
2 1 4 3

Algorithm: BruteForce
Distance: Spearman
Score: 1.333333

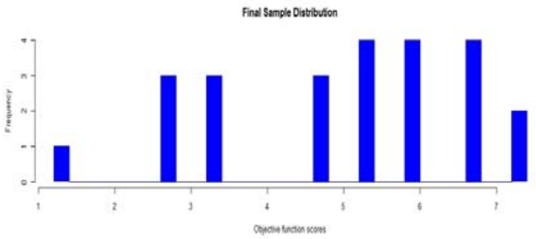

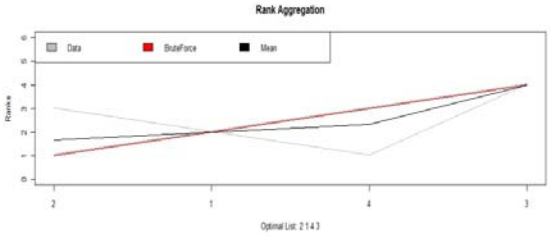

Rank aggregation without weights

x <- matrix(c
("4", "1", "2", "3",
"1", "2", "4", "3",
 "2", "1", "4", "3")
byrow=TRUE, ncol=4)
> (toplist <- BruteAggreg(x, 4))

The optimal list is:
1 2 4 3

Algorithm: BruteForce
Distance: Spearman
Score: 2

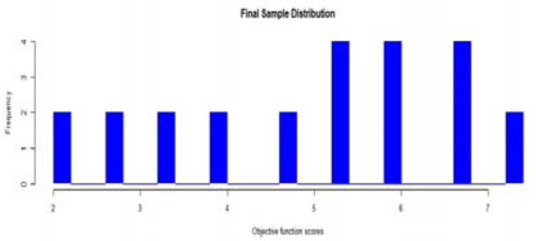

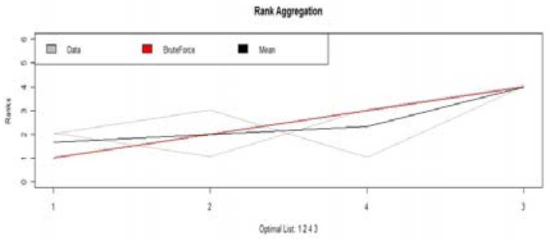

Gene references

1	*18S*
2	*B2M*
3	*HPRT1*
4	*SDHA*
